# Transcriptomic Signature and Growth Factor Regulation of Castration-Tolerant Prostate Luminal Progenitor Cells

**DOI:** 10.3390/cancers14153775

**Published:** 2022-08-03

**Authors:** Manon Baures, Emilia Puig Lombardi, Delphine Di Martino, Wail Zeitouni, Emeline Pacreau, Leïla Dos Santos, Charles Dariane, Florence Boutillon, Jacques-Emmanuel Guidotti, Vincent Goffin

**Affiliations:** 1 Institut Necker Enfants Malades, INSERM UMR-S 1151, CNRS UMR-S 8253, Université Paris Cité, F-75015 Paris, France; manon.baures@inserm.fr (M.B.); delphine.dimartino@gmail.com (D.D.M.); emeline.pacreau@inserm.fr (E.P.); leila.dos-santos@inserm.fr (L.D.S.); charles.dariane@inserm.fr (C.D.); florence.boutillon@univ-rennes1.fr (F.B.); jacques-emmanuel.guidotti@inserm.fr (J.-E.G.); 2 Bioinformatics Core Platform, Imagine Institute, INSERM UMR1163 and Structure Fédérative de Recherche Necker, INSERM US24/CNRS UAR3633, Université Paris Cité, F-75015 Paris, France; maria-emilia.puig-lombardi@institutimagine.org; 3 Genosplice, F-75014 Paris, France; wail.zeitouni@genosplice.com

**Keywords:** LSC^med^, luminal progenitor, Club/Hillock cells, CRPC, signature, organoid, EGFR, IGF1-R, MET, drug resistance

## Abstract

**Simple Summary:**

The shift from hormone-sensitive prostate cancer to castration-resistant prostate cancer (CRPC) has been hypothesized to be driven by prostatic luminal cells exhibiting castration tolerance, progenitor and tumor-initiating capacity. LSC^med^ cells that we recently isolated in a relevant mouse model of CRPC fulfil these three criteria. Using various bioinformatic pipelines, we here demonstrate that LSC^med^ cells match Club/Hillock cells recently identified in human prostate and prostate cancer. We identified EGFR/ERBB4, IGF-1 and MET pathways as key regulators of LSC^med^ cell progenitor and growth properties. We also demonstrate, for the first time in primary cultures of castration-tolerant prostatic progenitor cells, that the functional redundancy of these growth factor pathways confers to these cells the ability to bypass receptor-targeted pharmacological inhibition. Given the failure of EGFR- and MET-targeted monotherapies in CRPC patients, our data further support LSC^med^ cells as a relevant preclinical model to study the cellular and molecular mechanisms driving CRPC.

**Abstract:**

**Background:** The molecular and cellular mechanisms that drive castration-resistant prostate cancer (CRPC) remain poorly understood. LSC^med^ cells defines an FACS-enriched population of castration-tolerant luminal progenitor cells that has been proposed to promote tumorigenesis and CRPC in *Pten*-deficient mice. The goals of this study were to assess the relevance of LSC^med^ cells through the analysis of their molecular proximity with luminal progenitor-like cell clusters identified by single-cell (sc)RNA-seq analyses of mouse and human prostates, and to investigate their regulation by in silico-predicted growth factors present in the prostatic microenvironment. **Methods**: Several bioinformatic pipelines were used for pan-transcriptomic analyses. LSC^med^ cells isolated by cell sorting from healthy and malignant mouse prostates were characterized using RT-qPCR, immunofluorescence and organoid assays. **Results**: LSC^med^ cells match (i) mouse luminal progenitor cell clusters identified in scRNA-seq analyses for which we provide a common 15-gene signature including the previously identified LSC^med^ marker *Krt4*, and (ii) Club/Hillock cells of the human prostate. This transcriptional overlap was maintained in cancer contexts. EGFR/ERBB4, IGF-1R and MET pathways were identified as autocrine/paracrine regulators of progenitor, proliferation and differentiation properties of LSC^med^ cells. The functional redundancy of these signaling pathways allows them to bypass the effect of receptor-targeted pharmacological inhibitors. **Conclusions**: Based on transcriptomic profile and pharmacological resistance to monotherapies that failed in CRPC patients, this study supports LSC^med^ cells as a relevant model to investigate the role of castration-tolerant progenitor cells in human prostate cancer progression.

## 1. Introduction

Localized prostate cancer is successfully treated in more than 85% of cases by surgery (radical prostatectomy), external beam radiation therapy or brachytherapy [1]. In patients with metastatic disease, androgen-deprivation therapy (ADT) is the gold standard treatment. ADT will induce cancer regression, relieve symptoms and prolong survival. However, after an initial response to ADT, all patients with prostate cancer will ultimately progress to castration-resistant prostate cancer (CRPC). Despite recent progress in the clinical management of those patients [2], metastatic CRPC remains a lethal disease, and paracrine/autocrine androgen synthesis is thought to be one of the mechanisms of resistance to castration [3,4]. The identification of the cell(s) that drive cancer relapse and of the molecular pathways they use to promote tumor regrowth is needed. These cells should combine at least three properties: (1) castration tolerance, as they survive ADT; (2) luminal features, as the vast majority of hormone-sensitive prostate cancers (HSPC), from which CRPC arises, exhibit a luminal phenotype; (3) stemness, as they are assumed to rapidly regrow to form a tumor displaying phenotypic heterogeneity.

We recently discovered, isolated and profiled in mouse prostate an unprecedentedly defined population of non-secretory luminal cells matching these three criteria [5,6,7,8]. We named these cells LSC^med^ according to their FACS profile (Lin^−^/Sca-1^+^/CD49f^med^) using stem cell antigen-1 (SCA-1) and CD49f (integrin α6) as cell surface markers [6]. These cells were identified by others as SCA-1^+^ luminal cells [9]. Cytokeratin 4 (CK4) was validated as a specific protein biomarker of LSC^med^ cells on prostate sections from various mouse models [5,10]. The intrinsic castration tolerance of these cells was shown by their increased prevalence in prostates from castrated versus intact mice [5,9], and by their insensitivity to enzalutamide [9], a second-generation antiandrogen drug efficient at the HSPC and CRPC stages of the disease [11,12,13]. The stem/progenitor properties of LSC^med^ cells have also been widely assessed by us and others through their enriched capacity versus mature luminal cells to form spheres and organoids in vitro, to self-renew, and to generate glandular structures when engrafted into host mice [5,6,9]. Exhaustive description of LSC^med^ cell properties is provided in a recent review article [8].

The involvement of LSC^med^ cells in prostate cancer is supported by several observations in preclinical models for a review, Ref. [8]. While they are rare in healthy prostates (~5% of epithelial cells), LSC^med^ cells represent up to 80% of epithelial cells in prostate tumors driven by prostate-specific deficiency of the tumor suppressor gene *Pten* (mice are hereafter called Pten-null) [5,7]. In prostates of castrated Pten-null mice, LSC^med^ cells remain highly prevalent, and the detection of large clusters of CK4^+^/Ki67^+^ cells revealed that some LSC^med^ cells not only survive castration, but also proliferate [5]. Finally, FACS-enriched Pten-null LSC^med^ cells generate invasive tumors when engrafted into host mice [5]. Together, these data suggest that, in mice, LSC^med^ luminal progenitor cells actively contribute to prostate cancer progression towards CRPC. Based on these observations, the goal of this study was to address the relevance of LSC^med^ luminal progenitor cells to model human prostate cancer progression, including the identification of actionable targets able to interfere with this process.

Within the past few years, several groups published single cell (sc) atlases of the adult prostate based on RNA sequencing (scRNA-seq) data. These studies involved WT mice [10,14,15,16,17], *Pten*-deficient mice [18], and human specimens of healthy prostate [10,15,16,17,19] and prostate cancer [20,21]. All mouse studies identified one computerized cluster of non-secretory luminal cells referred to as ‘luminal progenitors’ based on their enrichment in stemness-related transcripts. The human studies identified one or two populations of non-secretory luminal-like cells called Club and Hillock based on their molecular similarity with eponymous epithelial progenitor cell types described in the lung [19]. Noteworthy, cancer-associated Club cells has been recently identified in prostate cancer specimens [20]. The correspondence of all these progenitor-like luminal cells has not been investigated beyond the qualitative overlap of a few markers. Using bona fide bioinformatic approaches, we here demonstrate that, in both healthy and cancer contexts, FACS-enriched LSC^med^ cells largely overlap with in silico-defined clusters of mouse luminal progenitor cells and human prostatic Club/Hillock cells. We provide a common 15-gene signature of castration-tolerant mouse luminal progenitor cells that should help to track these cells in preclinical models of prostate cancer in order to delineate their fate during cancer progression.

The second aim of this study was to investigate the regulation of LSC^med^ cell proliferation by growth factors present in the prostatic microenvironment. Bioinformatic search for autocrine/paracrine ligand–receptor pairs identified receptors of the epidermal growth factor receptor (EGFR/ERBB4), insulin-like growth factor-1 receptor (IGF-1R) and MET pathways as top candidates. Strikingly, monotherapies targeting these receptors were disappointing in metastatic CRPC patients [22,23,24,25,26,27,28,29,30,31]. The interactive crosstalk between MET and EGFR family members is well documented and has been raised as a mechanism of resistance to targeted monotherapies in other cancers [32,33,34]. Using the acknowledged organoid assay to monitor the regulation of LSC^med^ cell progenitor and growth properties by these growth factor pathways, we here document for the first time that castration-tolerant prostatic progenitor cells are able to evade EGFR, MET and IGF-1R pharmacological blockade. Together, our observations provide a strong rationale for the involvement of luminal progenitor cells in tumor progression observed in CRPC patients.

## 2. Materials and Methods

**Animals**. Pten-null mice were generated by breeding Pten^flox/flox^ female mice with Pb-Cre4 transgenic males on the C57BL/6J background as previously described [5]. Experiments were performed using 8 to 11-month-old mice, i.e., when aggressive malignant phenotypes were well established. Non-transgenic C57BL/6J littermates were used as controls and are referred to as WT animals. Colonies were housed in conventional health status, on a 12/12 h light/dark cycle with normal chow diet and water provided ad libitum. Prostate samples were obtained by microdissection immediately after sacrifice by cervical dislocation. Animal experiments were approved by the local ethical committee for animal experimentation (APAFIS#1427-2017121915584941).

**Prostate cell subpopulation sorting by FACS**. The procedures for cell sorting were performed as previously described [5,35]. Isolated cells (basal, luminal, LSC^med^ and stromal) were stained for FACS on ice for 30 min. Antibodies (eBioscience) used for FACS were fluorescein isothiocyanate-coupled lineage (Lin) antibodies (anti-CD31, CD45 and TER-119), phosphatidylethanolamine-Cyanine7-coupled anti-EpCAM, phosphatidylethanolamine-coupled anti-CD49f (integrin alpha-6) and allophycocyanin-coupled anti-Sca1 (lymphocyte antigen 6A-2/6E-1). Dead cells were colored with SYTOX blue. Cell sorting was performed on a BD FACS Aria III. Lin antibodies were used to deplete hematopoietic, endothelial and immune cells and EpCAM antibody was used to separate epithelial versus stromal cells. CD49f and SCA-1 markers were used to select basal cells, luminal cells and LSC^med^ cells in the EpCAM^+^ gate, and the stromal cells in the EpCAM^neg^ gate. Sorted cells were collected in DMEM medium, supplemented with 50% FBS, glutamine, and penicillin-streptomycin, or in RA1 Lysis Buffer (Macherey-Nagel, Düren, Germany) to perform RNA extraction as described in the manufacturer’s protocol.

**Reverse Transcription-Quantitative PCR (RT-qPCR)**. RNA extraction was performed with the Nucleospin RNA XS (Macherey-Nagel, Düren, Germany), as described in the manufacturer’s protocol. The reverse transcription was performed using the SuperScript™ VILO™ cDNA Synthesis Kit (Invitrogen) described protocol.

For qPCR, iTaq Universal SYBR Green Supermix (Promega, Madison, WI, USA) was used, and reactions were run on a qTower 2.0 real-time thermal cycler (Analytik Jena). Primers are listed in Table S1. Expression data obtained using Pten-null mouse prostates are presented as 2^−ΔΔCt^ normalized to WT mouse values (Ct represents the cycle threshold at which amplified cDNA is detected; the higher the Ct, the lower the gene expression).

**3D organoid culture**. We used the reference protocol described by Clevers’ lab [36], in which EGF is used as the growth factor. In our study, various growth factors were substituted for EGF. Culture media, additives, growth factors and drugs are listed in Table S2.

LSC^med^ cells sorted from Pten-null mouse prostates were plated in triplicate on a Low Growth Factor-containing Matrigel (Corning) layer in a 96-well plate (Falcon) in the presence or not of growth factors, according to the culture condition. After 1 day of incubation, the medium was removed and cells were covered by a new layer of Matrigel in order to perform 3D culture. The organoid-forming capacity did not differ from the efficacy obtained using 3D droplet culture. Drugs were added after 1 day of culture. Medium was changed every 2 days. After 10 days of Matrigel embedding, organoids were fixed in 4% PFA, and photos were taken with a 4× objective under a M5000 EVOS inverted microscope in order to cover the entire surface of the well. Counting and surfacing were performed on Fiji Software by manually surrounding the organoid surface. The number of organoids obtained in the various experimental conditions was normalized to the mean value obtained in the EGF-containing medium.

**Organoid agarose embedding**. After PFA fixation, all wells were combined, according to the culture conditions. Matrigel pellets were collected in BD Cell Recovery solution (Corning) and placed into ice during 30 min to 1 h, according to the number of pooled wells, in order to depolymerize the Matrigel. When the organoids started to settle at the bottom of the collecting tube, the supernatant was removed and pellets were resuspended in 2% low melting agarose. After solidification, the agarose pellets were transferred to 70% EtOH in order to perform paraffin wax protocol.

**Immunofluorescence** (IF). All samples were fixed in 4% PFA, paraffin wax-embedded, and sections underwent heat-induced antigen retrieval in citrate buffer at pH 6 (95 °C, 30 min). IF was performed as described previously [5] using antibodies directed against CK4 (1/150, BSM-52062R, ThermoFisher Scientific), CK5 (1/150, 905901, Biolegend) and CK8 (1/100, AB_531826, DSHB), KI-67 (1/150, RBK027-05, Diagomics) and E-cadherin (1/100, 610182, BD Transduction). Nuclei were stained with Hoechst dye. Samples were analyzed with a 40× objective under an Apotome 2 (Zeiss) microscope. Cell size was measured on E-cadherin-stained slides (350 cells per condition) by using the Cellpose 1.0 plugin associated with QuPath software.

**In silico identification of candidate pathways**. Cellular interaction prediction was performed using CellPhoneDB [37] with default parameters applied to three transcriptomic datasets for each sorted cell population of WT mouse prostates [5]. Human gene orthologs were used to take advantage of the CellPhoneDB ligand–receptor database (human). The *p*-value threshold to consider an interaction as significant was set to 0.05 (*p*-value ≤ 0.05). Significant predicted interactions involved in the positive regulation of cell proliferation (GO:0008284) having the receptor expressed by LSC^med^ cells and a soluble mitogenic ligand (irrespective of the cell compartment of origin) were selected.

**scRNA-seq data retrieval**. Data re-analyzed as part of this study were retrieved from the Gene Expression Omnibus (GEO) and The National Omics Data Encyclopedia (NODE) public databases (Table 1). For mouse scRNA-seq data, we only retained samples taken from intact (i.e., non-castrated) mice. Data retrieved from GEO repository GSE151944 were available as MULTI-seq sample barcodes; these were demultiplexed using the *MULTIseqDemux* function implemented in Seurat [38]. For datasets GSE145861 and GSE145865 [17], the data from the prostate and urethral regions were aggregated during the analysis step. For dataset GSE164858 [18] we retained the vehicle-treated sample only. Finally, data retrieved from the OEP000825 repository were raw fastq files which were processed (read alignment, generation of feature-barcode matrices) with Cell Ranger (10× Genomics) prior to any data analysis step.

Human healthy prostate data were readily available from two studies [16,17] and for each of these datasets, the different donor samples were aggregated during the analysis step. In addition, we retrieved scRNA-seq data for 11 prostate cancer patients (3 biopsies and 8 radical prostatectomies that were split into 27 samples and that we merged prior to integration) from the study described in Ref. [20], and data for 13 prostate tumor samples (that were initially merged into a single data matrix) from Ref. [21].

**Data analysis**. All datasets were processed using the same analytical pipeline. Low quality cells were filtered out by consecutively filtering each sample individually (i.e., prior to any aggregation step) based on unique molecular identifiers (UMI) counts, percentage mitochondrial content and number of genes (in that precise order). No filter was applied on the percentage of ribosomal gene content. Filtering was performed as previously described [17,19]: filter thresholds were chosen dynamically for samples based on the distribution of each parameter, with code adapted from sc-TissueMapper (v2.0.0) [39]. Upper and lower filters were applied on UMIs (the lower bound of the UMI filter was strictly set to 200 whilst the *RenyiEntropy* thresholding technique was applied to determine the upper bound after binning the data), while the percentage mitochondrial content had only an upper filter (abnormally high percentages of mitochondrial content were determined using the *Triangle* filter on binned data) and feature number had only lower filters (determined using the *MinErrorI* filter on binned data). *RenyiEntropy*, *Triangular*, and *MinErrorI* thresholding were applied using functions from the autothresholdr (v1.3.9) R package. For a given dataset, if multiple samples were available, samples were aggregated by normalizing with the *sctransform* (version 0.3.2) method and using Seurat’s reciprocal principal component analysis (PCA) method. Furthermore, cells displaying high stress signatures associated with the tissue dissociation experimental step were removed as described [19]: aggregated cells were scored for stress using Seurat’s *AddModuleScore* method and a mouse (or its human equivalent) gene set enriched for stressed cells earlier described [40]. Finally, PCA was performed on the data and graph-based clustering was performed using the principal components representing 90% of the associated cumulative variance.

**LSC^med^ score calculation**. LSC^med^-specific genes (*n* = 111 genes, assayed in WT mice) were retrieved from Ref. [5]. The LSC^med^ similarity score was based on the calculation of the average mRNA levels of the 111 signature genes for each single cell (to which is subtracted the averaged mRNA levels of 50 randomly chosen control genes) using Seurat’s *AddModuleScore* method. For calculation in the human prostate datasets, mouse stable gene IDs were matched to human stable gene IDs, retaining one-to-one ortholog matches. The mouse–human orthology table was generated with Ensembl BioMart.

**Marker gene identification**. Differentially expressed genes for the identified cell subpopulations were determined using Wilcoxon rank sum tests on genes present in at least 20% of cells in the population of interest, only retaining positive gene markers. Testing was limited to genes which showed, on average, at least 0.2-fold difference (on a log-scale) between the different groups. Finally, genes displaying an adjusted *p*-value inferior to 5% (*P_adj_* < 0.05) were retained.

**Pathway enrichment analysis**. The GO-BP (Biological Process) pathways enrichment analysis was performed using the GSEA (Gene Set Enrichment Analysis) software [41] with the Molecular Signatures Database collection on the identified marker gene sets for LSC^med^-like cell subpopulations. Significantly enriched pathways were selected based on FDR q-values (*p*-value adjustment for multiple hypergeometric tests), with the cutoff FDR < 5%.

**Statistical analyses**. The specific statistical tests performed are described in the legends to Figures. In summary, Analysis of Variance (ANOVA) tests were used to evaluate differences among three or more groups. Depending on the number of factors tested, one- or two-way ANOVAs were used. Simple or multiple comparisons were performed with Dunnett’s or Tukey’s post hoc tests, respectively. One, two, three or four symbols illustrating significance represent *p* values < 0.05, <0.01, <0.001 and <0.0001, respectively. A value of *p* < 0.05 was used as significance cutoff for all tests. Error bars represent S.E.M. All analyses were performed using GraphPad Prism version 9.00 for Windows (GraphPad Software, San Diego, CA, USA).

## 3. Results

### 3.1. Relevance of LSC^med^ Cells in Prostate Pathophysiology

#### 3.1.1. A Common Transcriptomic Signature Defines Mouse Prostate Luminal Progenitor Cell Clusters Identified in scRNA-Seq Studies

All mouse scRNA-seq studies [10,14,15,16,17] identified one population of non-secretory luminal cells that was referred to as prostate luminal progenitors in all but one study [17]. However, different nomenclatures were used (Lum D [14], Lum 2 [15], Lum P [16], Lum C [10] and Ur Lum [17]) and the overlap between these luminal progenitor cell clusters was not assessed using bona fide bioinformatic approaches. To address this question, we reanalyzed their publicly available scRNA-seq transcriptomic data (see Table 1 in Methods) to identify the top gene markers of luminal progenitors in each individual study and compare their expression level in each cell population across studies. Importantly, we applied the same analytical pipeline on count data retrieved for each of the studies, notably allowing us to identify and remove stressed cells from the analyses (see Data analysis in Methods) and enabling direct comparisons between the different datasets. Starting from 4624, 13,688, 5288, 34,444 and 90,345 single cells for each study (Table 1), we retained 1213, 5158, 2362, 19,503 and 45,432 single cells, respectively, after quality filtering, with a median of 2205 genes assayed per cell.

**Figure 1:**
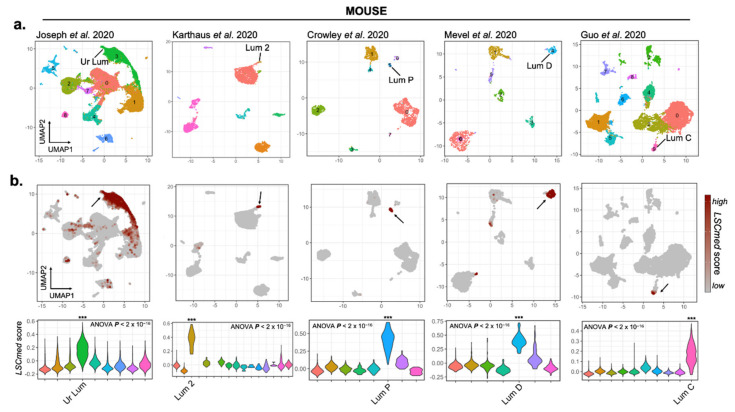
Transcriptomic similarity between FACS-enriched WT mouse LSC^med^ cells and luminal progenitor cell clusters identified by scRNA-seq analyses of WT mouse prostates. (**a**) UMAP projections based on linear dimensionality reduction by principal component analysis (PCA) for, from left to right, 45,432 [17], 5158 [15], 2362 [16], 1213 [14] and 19,503 [10] single-cell transcriptomes. (**b**) In each study, a single subpopulation matched LSC^med^-like cells [5], as shown by high calculated LSC^med^ gene signature scores (see LSC^med^ score calculation in Methods). The violin plots show the calculated LSC^med^ scores per cluster and per study (***, Tukey’s multiple comparisons of means *P_adj_* < 0.001 for all pairwise comparisons performed).

Subsequently, clustering was followed by dimensional reduction for visualization using UMAP (Uniform Manifold Approximation and Projection) plots, which depict cell populations with distinct transcriptional signatures (Figure 1a). For each dataset, we identified between 7 and 14 in silico-computed clusters expressing different phenotypic markers (epithelial, immune and stromal cells) as described [10,14,15,16,17], which were used to match previously labeled sub-populations of interest to our re-analyzed datasets (Figure 1a). In particular, we were able to recapitulate the Lum D [14], Lum 2 [15], Lum P [16], Lum C [10] and Ur Lum [17] subsets of non-secretory luminal progenitor cells as clear clusters based on marker gene identification for this particular cell subset (Table S3). Gene ontology analysis showed that this cell cluster is enriched for epithelial cell differentiation and tissue development, including cell proliferation and migration (Figure S1). This is in good agreement with the enrichment in progenitor properties expected for this particular cell cluster.

**Figure 2:**
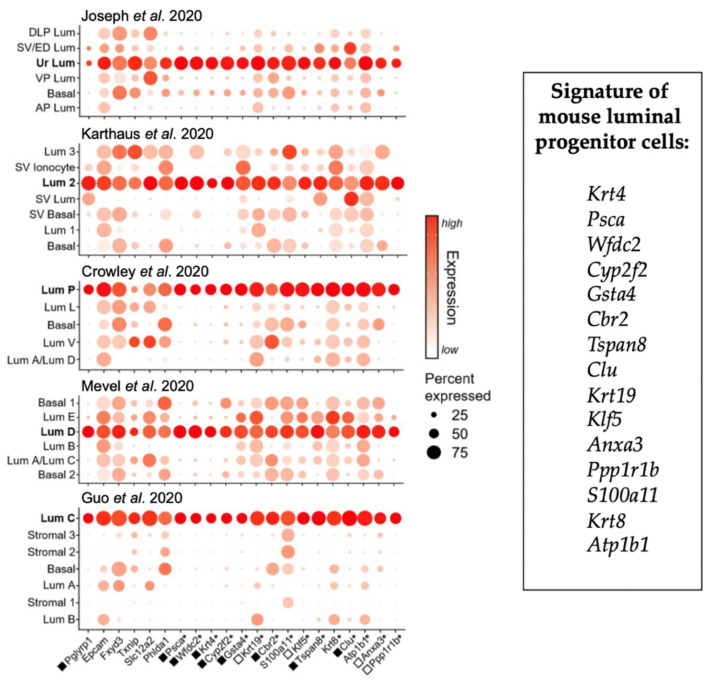
Common markers of luminal progenitors identified by scRNA-seq study of WT mouse prostate. Dot plot representation of marker genes for luminal progenitor cell subpopulations found in the five scRNA-seq studies discussed in the text [10,14,15,16,17], showing their relative expression across all detected clusters. Each dot depicts both detection rate and average gene expression in detected cells for a gene in a cluster. Darker red colors indicate higher average gene expression, and a larger dot diameter indicates that the gene was detected in greater proportion of cells from the cluster. Stars (*) depict the 15 marker genes detected in all datasets that constitute the mouse luminal progenitor cell signature. Squares indicate marker genes that were significantly (*p* < 0.05) identified in the LSC^med^ cell signature using 1.5 (black) or 1.2 (white) fold-change [5]. The other genes were all expressed in LSC^med^ cells at similar levels as in basal and/or luminal cells, and therefore, they were not identified as LSC^med^ cell markers. AP, anterior prostate; DLP, dorsolateral prostate; ED, ejaculatory duct; Lum, luminal; LP, lateral prostate; SV, seminal vesicle; Ur: urethra; VP, ventral prostate.

Analysis of the marker genes reported for the luminal progenitor cluster in the various scRNA-seq studies (Table S3) allowed us to identify 21 different genes common to at least 4 studies, and 15 common to all studies (Figure 2). These included *Krt4*, *Psca*, *Clu*, *Wfdc2*, *Cyp2f2*, *Tspan8*, *Gsta4* and *Tacstd2*. Of note, TROP2 (encoded by *Tacstd2*) has also been used as a surface protein biomarker to enrich luminal progenitors by cell sorting [10,16,42]. This analysis demonstrates that the various luminal progenitor clusters identified by scRNA-seq exhibit a high degree of similarity and can be defined by a common phenotypic signature.

#### 3.1.2. LSC^med^ Cells Largely Overlap with Luminal Progenitor Cell Clusters Identified by scRNA-Seq in Healthy and Malignant Mouse Prostates

The transcriptomic signature of mouse luminal progenitor cells identified by scRNA-seq (Figure 2) contains many genes earlier defined as LSC^med^-specific, including *Krt4* [5]. This observation suggested that the ex vivo-enriched (LSC^med^) and in silico-defined luminal progenitor cell populations might overlap. To address this hypothesis, we sought for the enrichment of the LSC^med^ cell signature by calculating a score based on the average expression of its 111 genes in the various prostate cell populations identified in silico. As expected, significant enrichment of the LSC^med^ cell signature was observed in each scRNA-seq dataset for the sole population identified as luminal progenitors (Figure 1a,b). Similar enrichment of the LSC^med^ cell signature was also observed in the progenitor cell cluster identified as Lum C1 in mouse prostate tumors generated by the ablation of *Pten* selectively in luminal prostatic epithelial cells at adulthood [18] (Figure S2).

Altogether, these analyses indicate that LSC^med^ progenitor cells enriched by cell sorting largely overlap with the luminal progenitor cell cluster identified by scRNA-seq in both healthy and cancer contexts.

#### 3.1.3. LSC^med^ Cells Largely Overlap with Club and Hillock Cells of Human Healthy Prostate and Prostate Cancer

Club/Hillock cells of the human prostate [19] share typical markers with mouse LSC^med^-like luminal progenitors (e.g., *KRT4*, *TACSTD2* and *PSCA*), but the actual correspondence between these cell entities also remains elusive [15,16]. To address this question, we proceeded as above and sought for the enrichment of the mouse LSC^med^ cell signature in the various human prostate cell populations identified in silico. Using two scRNA-seq datasets from healthy prostate [16,17] and two from prostate cancer specimens [20,21], we applied the same analytical pipeline as described for the mouse scRNA-seq datasets to the retrieved human prostate data.

**Figure 3:**
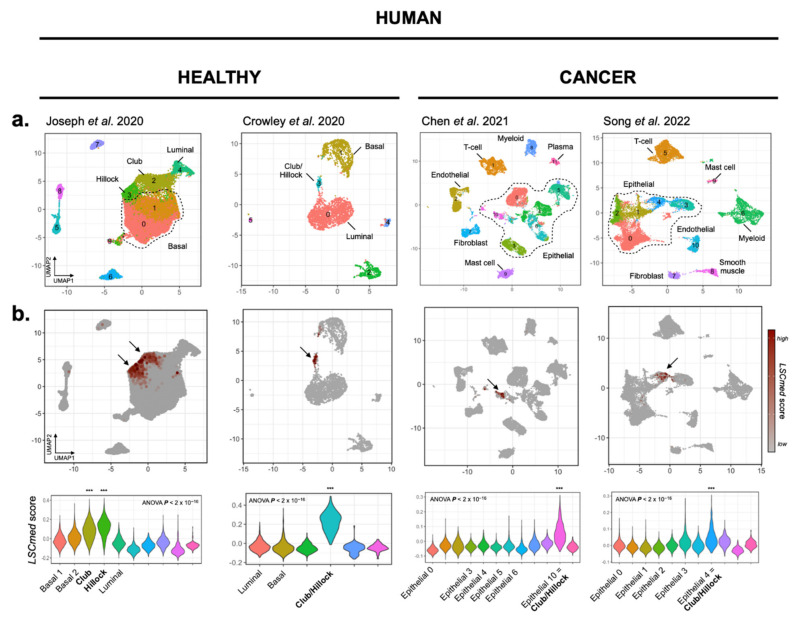
Transcriptomic similarity between FACS-enriched WT mouse LSC^med^ cells and Club and Hillock cell clusters identified by scRNA-seq analyses of human healthy prostates and prostate cancer. (**a**) UMAP projections based on linear dimensionality reduction by principal component analysis (PCA) for 28,759 [17] and 3352 [16] healthy prostate single-cell transcriptomes, as well as 24,203 [20] and 14,937 [21] prostate cancer single-cell transcriptomes. Club and Hillock cells could be identified in the Joseph et al. dataset, but a single Club/Hillock cluster was identified in all other datasets. (**b**) In each study, only Club and/or Hillock cell subpopulations matched LSC^med^-like cells, as shown by high calculated LSC^med^ gene signature scores. The violin plots show the calculated LSC^med^ scores per cluster and per study (***, Tukey’s multiple comparisons of means *P_adj_ <* 0.001 for all pairwise comparisons performed).

In the healthy prostate, we observed a significant enrichment of the LSC^med^ cell signature for the sole populations identified as Club/Hillock cells. Interestingly, while marker genes for both Hillock and Club cells were identified in a single cluster in the dataset of Crowley et al. [16], we were able to discriminate the two types of cells in the dataset of Joseph et al. [21] (Figure 3a,b, left panels). In addition, some LSC^med^ markers were predominantly expressed in Hillock cells (e.g., *KRT4*, *AREG*, *GSDMC*) or in Club cells (e.g., *S100A1*, *ATP10B*, *LTF*), while some were shared between both cell populations (e.g., *PSCA*, *ARL14*, *WFDC2*).

The analysis of biopsy and prostatectomy specimens from 11 [20] and 13 [21] untreated prostate cancer patients showed that in both datasets, the LSC^med^ signature was enriched in a single cluster of epithelial cells (Figure 3a,b, right panels). Moreover, this cell population expressed typical gene markers of the Club/Hillock cells described in healthy prostates (e.g., *KRT13*, *KRT4*, *MMP7*, *PIGR*, *SCGB3A1*, *LCN2*, *CP*). This cluster was identified in each individual patient, albeit at highly variable ratio ranging from 1% to 25% of the epithelial cell pool [21]. The mutational status of *PTEN* was not available in these two studies.

Altogether, the full transcriptomic profile comparisons reported in this first section provide unbiased evidence that mouse prostate LSC^med^ luminal progenitor cells enriched by cell sorting largely overlap with luminal progenitor clusters identified in mouse prostates by scRNA-seq, and correspond to Club/Hillock cells recently described in the human prostate using the same approach. Importantly, the LSC^med^ cell molecular identity was conserved in both healthy and malignant contexts.

### 3.2. Identification of Candidate Pathways Promoting LSC^med^ Cell Proliferation

#### 3.2.1. In Silico Identification of Ligand–Receptor Pairs

**Figure 4:**
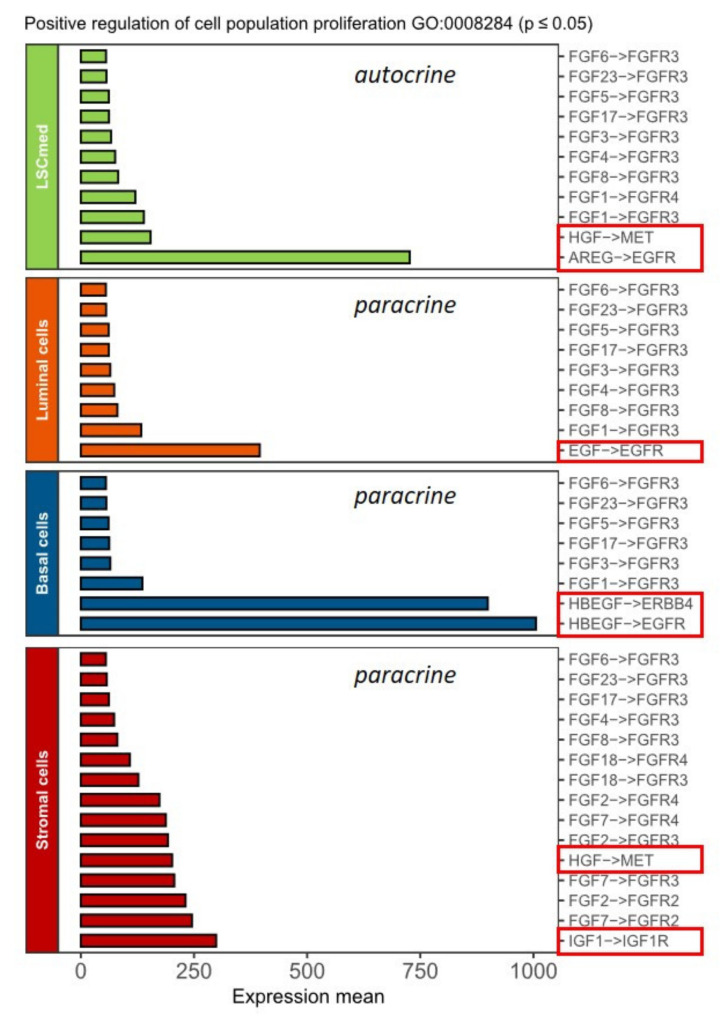
In silico prediction of growth factors regulating LSC^med^ cell proliferation. Bar diagram representing the significant ligand–receptor interactions identified by CellPhoneDB analysis applied to transcriptomic data of epithelial (LSC^med^, luminal, basal) and stromal cells of WT mouse prostate [5]. Only interactions involving receptors expressed by LSC^med^ cells and secreted ligands expressed by any of the four prostate cell compartments are reported. Red boxes identify interactions that have been experimentally challenged in this study (Ligand→Receptor). *Expression Mean = mean(mean(Receptor, compartment X), mean(Ligand, compartment Y))*.

To identify regulatory pathways of LSC^med^ cell proliferation, we sought for potential cell–cell interactions. To that end, the transcriptomes of WT LSC^med^, basal, luminal and stromal cell compartments that we earlier reported [5] were analyzed using CellPhoneDB ligand–receptor database [37]. Candidate interactions were filtered to select (i) receptors expressed by LSC^med^ cells, (ii) receptor pathways acting as positive regulators of cell proliferation, and (iii) secreted (by opposition to membrane-bound) factors testable in cell culture experiments. Figure 4 represents the significant interactions involving receptors expressed in LSC^med^ cells and cognate secreted ligands expressed by any of the four prostate cell compartments, encompassing both autocrine and paracrine candidates.

Amphiregulin (AREG)/EGFR was identified as the most significant autocrine ligand–receptor interaction in LSC^med^ cells. AREG is one of the over-expressed genes of the LSC^med^ cell signature [5], and the EGFR pathway has been reported to promote both prostatic cell proliferation [43,44] and stemness [45,46,47]. These findings support this pathway as a relevant autocrine regulator of LSC^med^ cells in vivo. The EGFR has multiple ligands, many of which are also present in the prostatic microenvironment. Accordingly, the two most significant paracrine interactions involved HB-EGF and EGF, two other EGFR ligands originating from basal and luminal cells, respectively. HB-EGF is also an agonist of ERBB4, another member of the EGFR/ERBB family [48]. In the stromal compartment, several paracrine growth factors exhibiting lower significance were identified, the top of which involved insulin-like growth factor (IGF)-1. MET, the hepatocyte growth factor (HGF) receptor, might also be activated in LSC^med^ cells via autocrine and paracrine (stromal) regulation. This analysis also identified several members of the fibroblast growth factor (FGF) family as potential candidates. However, both the lower predicted intensity of these interactions and the probable high degree of redundancy between the multiple FGFs (*n* = 9) and FGFRs (*n* = 3) identified in this analysis (Figure 4) led us to focus our study on the top candidate receptors, i.e., EGFR, ERBB4, MET and IGF-1 receptor (IGF-1R). As expected, expression of these receptors could be detected in the in silico-defined mouse and human LSC^med^-like cell populations (Figure S3a). Overall, there was a fair correlation between both species regarding the expression patterns of receptors (Figure S3a) and ligands (Figure S3b).

#### 3.2.2. Expression of Ligand–Receptor Pairs in WT and Pten-Null Mouse Prostate Cells

To validate the hypotheses generated by CellPhoneDB analyses, we first experimentally assessed the expression of the various ligand–receptor pairs in WT prostates, using RT-qPCR as earlier reported [5]. Expression of the four receptors of interest in LSC^med^ cells was detected with Ct < 30 (Figure S4a). All ligands but *Hbegf* were also detected with Ct < 30 in bulk prostate (not shown). Although not identified in silico, tumor growth factor α (*Tgfa*) was included in these experiments since this alternative EGFR ligand was proposed to be an autocrine promoter of prostate cancer progression [49,50,51]. In agreement with CellPhoneDB predictions, EGFR ligands were mainly expressed by epithelial cells, in particular luminal (*Egf*) and LSC^med^ (*Areg*, *Hbegf*, *Tgfa*) cells. *Igf1* was mainly expressed in the stromal compartment, and *Hgf* in both epithelial and stromal cells (Figure S4b). Co-expression of ligand–receptor pairs in LSC^med^ cells (Figure S4a,b) supports autocrine signaling for at least some of these growth factors.

**Figure 5:**
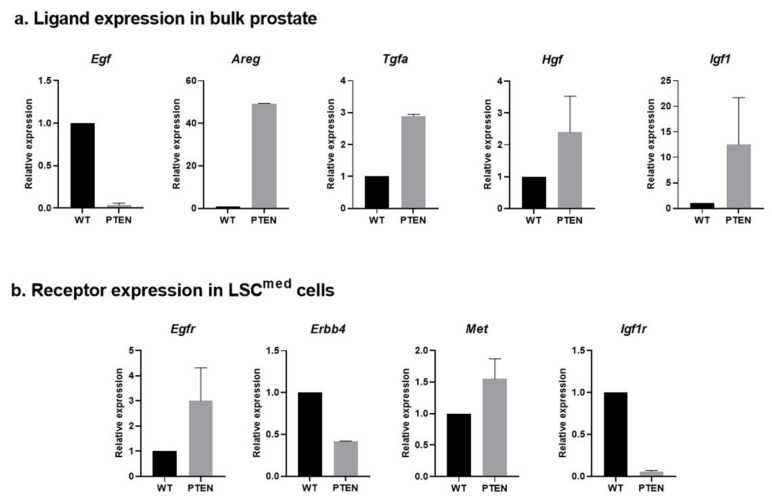
Relative expression of EGFR/ERBB4, MET and IGF-1R ligands (**a**) and receptors (**b**) in Pten-null prostates versus WT prostates. Expression of *Egfr*, *Erbb4*, *Met* and *Igf1r* in sorted LSC^med^ cells and of their ligands in bulk prostate was determined by RT-qPCR. Data obtained from 2 (**a**) and 3 (Pten-null) to 15 (WT) animals (**b**) are presented as 2^−ΔΔCt^ normalized to WT mouse values.

We then monitored the expression of these ligand–receptor pairs in Pten-null prostates. Expression data relative to WT prostates are shown in Figure 5a (ligand expression in bulk prostates), Figure S5 (ligand expression in sorted cell compartments) and Figure 5b (receptor expression in LSC^med^ cells).

Expression of all ligands but *Egf* was increased by 2- to >40-fold in bulk Pten-null compared to WT prostate (Figure 5a and Figure S5). The lower levels of *Egf* expression in Pten-null prostates result from the virtual loss of luminal cells where this growth factor is normally expressed (Figure S4b). The concomitant increase in *Areg* expression in Pten-null prostates (Figure 5a and Figure S4b) suggests the occurrence of a switch from EGF to AREG signaling during tumorigenesis. Additionally, the increased expression of *Hbegf*, *Tgfa*, *Hgf* et *Igf1* in basal and/or stromal cells at the expense of LSC^med^ in Pten-null samples (Figure S5) suggests that paracrine signaling involving these growth factors may increasingly contribute to LSC^med^ cell amplification during tumorigenesis. Finally, although *Igf1r* expression was reduced in Pten-null compared to WT LSC^med^ cells (Figure 5b), it remained detected at Ct < 30, i.e., in the same order of magnitude than the three other receptors in Pten-null prostate. The concomitant 10-fold increased expression of *Igf1* in Pten-null versus WT prostates (Figure 5a) argues for the persistence of IGF-1R signaling during tumorigenesis. Accordingly, Pten-null LSC^med^ cells were highly responsive to IGF-1 stimulation in vitro (see below).

### 3.3. Functional Regulation of LSC^med^ Cells by EGFR, ERBB4, MET and IGF-1R Signaling

The data presented in Section 3.2 predict EGFR/ERBB4, MET and IGF-1R signaling as relevant regulators of LSC^med^ cell proliferation, and suggest that the enrichment of their ligands in the microenvironment of Pten-null mouse prostates might contribute to the amplification of the LSC^med^ cell compartment observed in prostate tumors of these mice [5]. In this section, we aimed to experimentally assess these pathways as functional regulators of LSC^med^ cells.

Organoids are a self-organizing 3D culture system derived from pluripotent stem/progenitor cells [52]. This assay has been successfully applied to support the existence of luminal progenitors in mouse and human prostates, including LSC^med^ cells, and to characterize their properties [9,53,54]. The number, size and cellular composition of organoids generated from a given cell subset are considered to reflect their stem/progenitor, proliferative and differentiative properties, respectively [55,56]. It has also been used to determine the contribution of specific factors (e.g., androgens) or drugs to any of these properties [53,57]. Therefore, the organoid assay was appropriate to address whether the various growth factors of interest regulate these properties in LSC^med^ luminal progenitor cells.

#### 3.3.1. Regulation of Pten-Null Mouse LSC^med^ Cells by Growth Factors

The culture medium defined by the group of Clevers to generate organoids from mouse prostate progenitor cells contains EGF as growth factor [36,53]. In this medium, we observed that the organoid-forming capacity of LSC^med^ cells sorted from Pten-null mouse prostates was ~4%. This is very similar to what has been observed by us and others for WT mouse LSC^med^ cells (our unpublished data), WT SCA-1^+^ luminal progenitor cells [9] (that are equivalent to LSC^med^ cells; see Ref. [8]) and PROM1+ luminal progenitors enriched from Pten-null mice [58].

**Figure 6:**
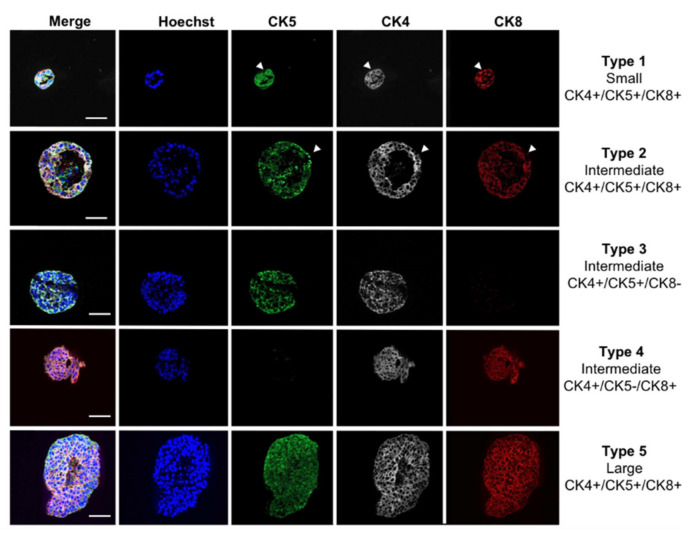
Immunofluorescence analysis of organoids generated from Pten-null mouse LSC^med^ cells. Immunofluorescence staining of CK5 (basal marker, green), CK4 (LSCmed marker, white) and CK8 (luminal marker, red; note that CK8 is expressed in both LSCmed and secretory luminal cells) and organoid size identified five histological types of organoids that were observed in DHT/EGF-containing culture medium (see text for description). Arrowheads label double-positive cells in type 1 and type 2 organoids otherwise positive for the three CKs tested. Hoechst was used to stain nuclei. The scale bar represents 100 µm.

Similar to prostatic glands in situ, organoids generated from WT LSC^med^ cells displayed a bilayered epithelium and frequently a lumen [9]. Based on lineage markers (not including CK4), four types of organoids were previously identified [9]. In agreement with the histology of tumoral glands of Pten-null mice, organoids generated in this study by LSC^med^ cells enriched from the latter mice exhibited a multilayered epithelium with reduced or absent lumen. Based on organoid size and CK4/CK5/CK8 protein expression detected by co-immunostaining, we identified five histological types of organoids represented in Figure 6. In type 1 and type 2 organoids, most cells expressed the three CKs analyzed while a few cells were only double positive (arrowheads on Figure 6). The other types of organoids exhibited a very homogeneous immunostaining pattern involving expression of two (types 3 and 4) or three (type 5) CKs in virtually all cells. Type 3 organoids exhibited a more basal phenotype (CK5+), and type 4 a more luminal (CK8+) phenotype. Irrespective of the type of organoid, we failed to detect fully differentiated luminal cells (CK4−/CK8+). This is reminiscent of the cell phenotypes observed in Pten-null prostate tumors in situ and in tumors grown from LSC^med^ engrafted into immunodeficient mice [5].

The histological diversity of organoids in both WT [9] and Pten-null (Figure 6) contexts presumably reflects that the FACS-enriched LSC^med^ cell pool contains cells exhibiting distinct differentiation capacities.

**Figure 7:**
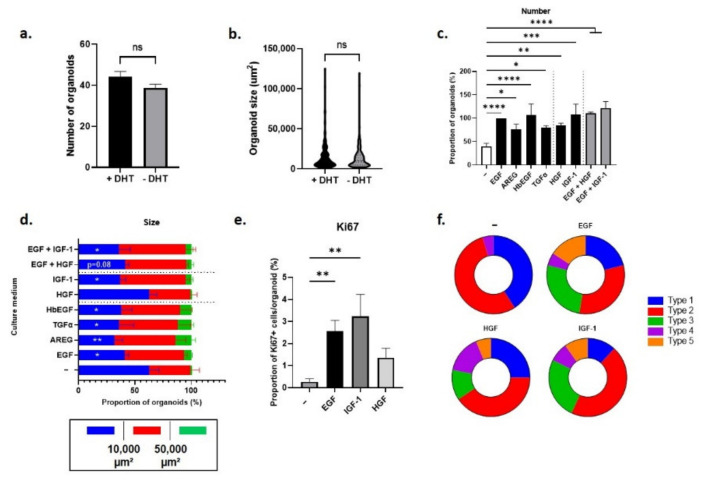
Impact of EGFR/ERBB4, MET and IGF-1R ligands on the number, size, cell proliferation and type of organoids generated from Pten-null LSC^med^ cells in DHT-containing medium. (**a**,**b**) Effect of DHT (1 nM) on the number (**a**) and size (**b**) of organoids generated by LSC^med^ cells FACS-enriched from *Pten*-null mouse prostates. (**c**,**d**) Effect of various growth factors substituted for, or combined to, EGF (as indicated) on the number (**c**) and size (**d**) of organoids measured at day 10 of culture. Organoid size is color-coded as follows: smaller than 10,000 µm^2^ (blue), between 10,000 and 50,000 µm^2^ (red), and higher than 50,000 µm^2^ (green). (**e**) The effect of various growth factors (as indicated) on cell proliferation in organoids was determined at day 10 by KI-67 IF analysis. (**f**) Schematic representation of the different types of organoids (described in Figure 6) that were generated in the presence of various growth factors (as indicated). See Table S2 for the concentrations of growth factors added to the culture medium. Data were obtained from 3 independent experiments each involving 1 or 2 animals. Statistical analyses were performed using t-test (**a**,**b**), one-way ANOVA followed by Dunnett’s post hoc test (**c**,**e**) or two-way ANOVA (**d**). * *p* < 0.05; ** *p* < 0.01; *** *p* < 0.001 and **** *p* < 0.0001 versus the condition without growth factor (noted as “-”).

Neither the number (Figure 7a), nor the size (Figure 7b) or the histology of organoids generated from Pten-null mouse LSC^med^ cells were markedly affected by the presence or the absence of dihydrotestosterone (DHT). This agrees with the intrinsic castration tolerance of LSC^med^ cells irrespective of the mouse model [5,9]. Accordingly, the effects of the various growth factors on the organoid-forming capacity of Pten-null mouse LSC^med^ cells were similar irrespective of the presence (Figure 7c,d) or the absence (Figure S6a,b) of DHT in culture medium.

As shown in Figure 7c, EGF withdrawal from culture medium drastically reduced the organoid-forming capacity of LSC^med^ cells compared to the EGF-containing medium used as the control condition in these experiments [36]. This was expected as this growth factor is critical for the formation of organoids from virtually all epithelial cell types [59], including the prostate [60]. The organoid-forming capacity could be fully restored by the addition of AREG, HB-EGF or TGFα (each individually), indicating that EGFR/ERBB4 signaling, more than EGF per se, is mandatory. The same rescuing effect was also observed by the addition of HGF or IGF-1, suggesting that the redundancy between EGFR/ERBB4, MET and IGF-1R signaling documented in other biological contexts also applies to the organoid-forming capacity of tumoral prostatic luminal progenitors. Accordingly, no additive effect was observed when HGF or IGF-1 were combined with EGF (Figure 7c).

Analysis of organoid size (Figure 7d) showed the accumulation of small-sized organoids (and the virtual absence of large ones) in the absence of growth factor, supporting the predicted role of EGF on LSC^med^ cell proliferation. This was confirmed by measuring the ratio of KI-67+ cells in organoids (Figure 7e). Alternative EGFR ligands and IGF-1 were able to fully substitute EGF for promoting organoid growth and LSC^med^ cell proliferation (Figure 7d,e), without affecting cell size (Figure S6c). In contrast, HGF mildly stimulated LSC^med^ cell proliferation (Figure 7e), and accordingly, it failed to restore the organoid size pattern observed with other growth factors (Figure 7d). HGF did not act as a proliferation inhibitor, however, as the combination of EGF and HGF achieved similar profile as with EGF alone (Figure 7d). Finally, while EGF, HGF or IGF-1 promoted the generation of the five histological types of organoids with a similar, albeit not strictly identical, distribution, types 3 and 5 organoids were not observed in the absence of growth factor, indicating the mandatory role of growth factor signaling for their generation (Figure 7f).

Together, these data indicate that EGFR/ERBB4, IGF-1R and MET signaling promote the progenitor and differentiation properties, and for the two formers, the proliferation of Pten-null mouse LSC^med^ cells, irrespective of the presence of androgens.

#### 3.3.2. Pharmacological Inhibition of EGFR/ERBB4 and MET Signaling in Pten-Null Mouse LSC^med^ Cells

**Figure 8:**
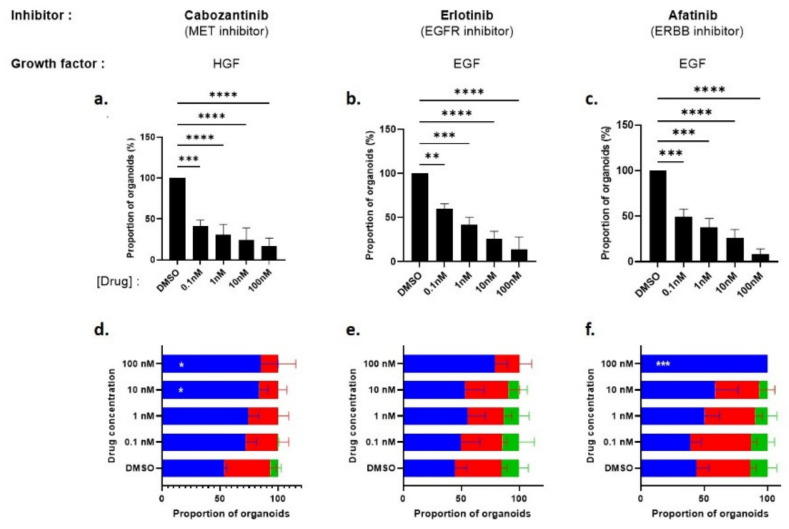
Impact of EGFR/ERBB4 and MET pharmacological inhibitors on the number and size of organoids generated from PTEN-null mouse LSC^med^ cells. Dose–response effect of Cabozantinib (**a**,**d**), Erlotinib (**b**,**e**) and Afatinib (**c**,**f**) on the number (**a**–**c**) and size (**d**–**f**) of organoids generated by LSC^med^ cells FACS-enriched from Pten-null mouse prostates. As in Figure 7, organoid size is color-coded as smaller than 10,000 µm^2^ (blue), between 10,000 and 50,000 µm^2^ (red), and higher than 50,000 µm^2^ (green). HGF (**a**,**d**) and EGF (**b**,**c**,**e**,**f**) were used as unique growth factor in the culture medium (each 50 ng/mL). Data were obtained from 3 experiments each involving 1 or 2 animals. Statistical analyses were performed using one-way ANOVA followed by Dunnett’s post hoc test (**a**–**c**) or two-way ANOVA (**d**–**f**). * *p* < 0.05; ** *p* < 0.01; *** *p* < 0.001 and **** *p* < 0.0001 versus the DMSO condition.

In order to further assess the effects reported above, the organoid-forming capacity (Figure 8a–c) and organoid size (Figure 8d–f) were analyzed after treatment of Pten-null mouse LSC^med^ cells with various acknowledged pharmacological receptor inhibitors: Erlotinib (a specific inhibitor of EGFR signaling [48]), Afatinib (an irreversible inhibitor of EGFR, ERBB4 and ERBB2) [32,48]) and Cabozantinib (a multitargeted tyrosine kinase inhibitor that has demonstrated activity against MET signaling [25]). These three inhibitors were used in DHT-supplemented culture media containing EGF (Erlotinib, Afatinib) or HGF (Cabozantinib) as the growth factor.

As shown in Figure 8a–c, the number of organoids decreased in a dose-dependent manner for all inhibitors, confirming that EGFR/ERBB4 and MET signaling regulate the organoid-forming capacity of Pten-null mouse LSC^med^ cells. The results obtained with Erlotinib and Afatinib were almost undistinguishable, arguing for the primary role of EGFR (versus ERBB2 and 4) in mediating organoid formation.

The organoid size parameter (Figure 8d–f) was less sensitive to the drugs, as only the highest dose of inhibitors achieved significant effects. According to the data shown in Figure 7d, HGF generated less large-sized organoids than EGF, and this effect was further dose-dependently increased by Cabozantinib. In contrast, the lower doses of Erlotinib and Afatinib had almost no effect on organoid size, and at the highest dose, Afatinib was more efficient than Erlotinib to prevent the formation of large organoids. This suggests that the latter property may also involve other receptors than EGFR, i.e., ERBB2 and/or ERBB4.

#### 3.3.3. Bypass Pharmacological EGFR and MET Signaling Inhibition by Alternative Growth Factors

**Figure 9:**
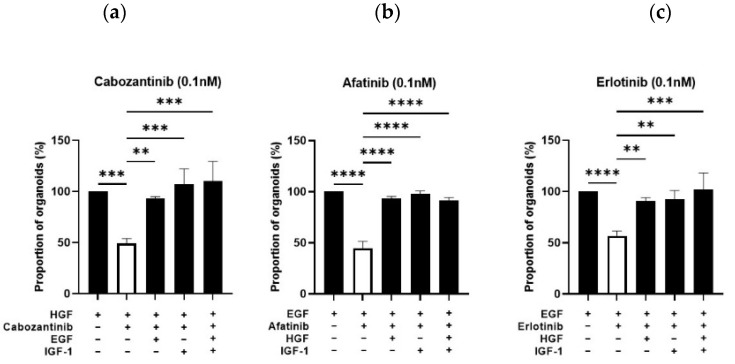
Growth factors rescue the ability of Pten-null mouse LSC^med^ cells to generate organoids in the presence of receptor tyrosine kinase inhibitors. (**a**) Effect of EGF and/or IGF-1 on the ability of LSC^med^ cells FACS-enriched from Pten-null mouse prostates to generate organoids in the presence of HGF and Cabozantinib. (**b**,**c**) Same as in panel a, with HGF and/or IGF-1 in the presence of EGF and Erlotinib (**b**) or Afatinib (**c**). See Table S2 for the concentrations of growth factors added to culture medium. These data were obtained from 3 experiments each involving 1 or 2 animals. Statistical analyses were performed using one-way ANOVA followed by Tukey’s multiple comparisons test. ** *p* < 0.01; *** *p* < 0.001 and **** *p* < 0.0001 versus the condition combining growth factor and inhibitor (white bars).

We showed in Figure 7 that alternative EGFR ligands (AREG, HB-EGF, TGFα) as well as alternative receptors (MET, IGF-1R) displayed similar efficiency to stimulate the capacity of Pten-null mouse LSC^med^ cells to generate organoids. To address whether this functional redundancy translated into the ability of these growth factors to bypass pharmacological inhibition of receptors, as shown in other biological contexts [32,33,34], different combinations of drugs and growth factors were tested. As shown in Figure 9a, inhibition of HGF-mediated MET signaling by Cabozantinib treatment (0.1 nM) was bypassed by the addition of EGF or IGF-1 as both growth factors fully rescued the organoid-formation capacity of LSC^med^ cells, without additive effect. The same rescuing effects were observed with HGF and IGF-1, alone and combined, when EGF-mediated signaling was inhibited by Erlotinib (Figure 9b) or Afatinib (Figure 9c). Similar rescuing effect was observed in the presence of 1000-fold higher concentration of Afatinib (100 nM; Figure S7), demonstrating that the effects observed in our experiments at lower concentration are not due to drug toxicity.

## 4. Discussion

Castration-resistant luminal progenitor cells are increasingly viewed as important contributors to prostate pathogenesis (for a review, see Ref. [8]). However, these cells remain poorly characterized: no consensus molecular identity has been established yet, and the description of their regulation by extracellular factors is scarce. Our study provides two steps forward regarding the understanding of these cells. First, using pan-transcriptomic comparisons, we identified a phenotypic molecular signature of luminal progenitors in the mouse, and we show that LSC^med^ cells largely overlap in silico-defined cell clusters referred to as ‘luminal progenitors’ in mice and ‘Club/Hillock cells’ in humans. Notably, the proximity between these cells was maintained in cancer contexts. Second, we identified EGFR/ERBB4, MET and IGF-1R signaling pathways as regulators of the organoid-forming capacity of LSC^med^ cells. The drug resistance offered by the functional redundancy of these pathways, here demonstrated for the first time in primary cultures of castration-tolerant luminal progenitors, echoes the failure of receptor-targeted monotherapies in CRPC patients. Together, our data support LSC^med^ cells, the prototypic prostatic luminal progenitor cells, as a relevant preclinical model of castration-tolerant cells assumed to contribute to prostate cancer progression towards CRPC.

The unsuspected complexity of the mouse prostate epithelium recently revealed by scRNA-seq studies [10,14,15,16,17,61] has stressed the need to carefully address the molecular definition of the cell cluster defined as prostatic luminal progenitor cells. To our knowledge, no bona fide bioinformatic analysis covering the various scRNA-seq data available has been performed to evaluate their actual correspondence across studies beyond the convergence of a few markers [16]. Furthermore, while CK4 was identified as a biomarker of this luminal progenitor cell cluster in some studies [10,14], the potential similarity with FACS-enriched LSC^med^ cells, of which CK4 is a specific marker [5], was ignored in all but one scRNA-seq report [17]. In the latter, however, the computed cell cluster was referred to as urethral luminal cells, which further complexifies our understanding of this prostatic cell cluster [17]. The present study involving pan-transcriptomic comparative analyses definitely confirms the molecular equivalence of the in silico-defined and ex vivo-enriched mouse luminal progenitor cell population, which is materialized by a robust 15-gene phenotypic signature encompassing information from five independent scRNA-seq studies. Although this signature includes *PSCA*, a typical stemness-related gene, only ~4% of these cells exhibit progenitor properties in functional stem cell assays including organoid formation and reconstitution assays (for a review, see Ref. [8]). The molecular identity of this particular cell subpopulation remains to be determined.

Our analyses also show the proximity of LSC^med^ cells with Club and Hillock cells of the human prostate [19]. In the healthy prostate, Club and Hillock cells are transcriptionally very similar, although the latter exhibit a more basal-like phenotype (e.g., *KRT5*^+^) than the former [15,17,19]. Typical LSC^med^ cell markers were found in both cell types, with some more specific to one or another cell population. This suggests that LSC^med^ cells is representative of both human cell entities. Although Club and Hillock cells are also enriched in *PSCA*, it is currently unknown whether they display increased stem/progenitor properties compared to mature luminal cells. Their molecular proximity with mouse LSC^med^ cells and with human pulmonary progenitor Club cells, which exhibit regenerative properties [62,63], argues in favor of this hypothesis, but this awaits experimental assessment.

Single cell RNA-seq studies of WT mouse prostates recently highlighted the transcriptional plasticity of epithelial luminal cells upon castration [14,15]. In particular, a transient and partial dedifferentiation of secretory luminal cells into a luminal progenitor-like profile was reported after mouse castration, and androgen addback reversed this transcriptional drift [15]. Similar findings were recently reported in human benign prostate hyperplasia (BPH). Treatment with 5α reductase inhibitors, which are used to reduce intraprostatic androgen receptor signaling in BPH cells, was associated with a shift of luminal cells towards a Club-like identity [64]. In keeping with this, we and others have demonstrated that mouse LSC^med^-like luminal progenitor cells are intrinsically castration tolerant in both healthy and cancer contexts [5,9,15]. In localized human prostate cancer specimens, several cellular states were identified in prostate epithelial cells, including one population referred to as “tumor-associated Club cells” assumed to be associated with prostate carcinogenesis [20]. Notably, this cluster showed transcriptomic proximity with CD38^low^ luminal progenitor cells previously identified as cancer-initiating cells associated with bad prognosis [8,65]. Based on these observations, the concept is emerging that androgen signaling deprivation may promote prostatic epithelial cell plasticity, thus leading to various cellular states among which the Club/Hillock/LSC^med^-like transcriptomic profile may constitute a functional hub for castration tolerance prior to molecular adaptations promoting CRPC. In this context, LSC^med^ cells enriched from Pten-null mice, an acknowledged mouse model of CRPC, represent a valuable preclinical model to address the molecular mechanisms driving the survival and expansion of tumor epithelial cells in ADT context.

Analysis of CRPC from castrated Pten-null mice revealed the presence of large clusters of KI-67-positive LSC^med^ cells [5], raising the question of the mechanisms promoting cell proliferation and tumor relapse. We here identified three growth factor families (EGFR/ERBB4, MET, IGF-1R) able to promote the organoid-forming capacity of Pten-null LSC^med^ cells. According to the intrinsic castration tolerance of these cells, similar effects were observed irrespective of the presence of DHT in culture medium, as previously reported for organoids grown from WT luminal progenitor cells cultured in classical EGF-containing medium [15]. Previous studies involving immortalized human prostate cancer cell lines have suggested the ability of these growth factors to stimulate stemness. In the DU145 cell line, EGFR signaling was shown to promote stem/progenitor properties the via ERK signaling [45] and this required SOX2 [47], a key gene for neuroendocrine differentiation of Pten-null LSC^med^-like cells [66]. In the 22Rv1 cell line, HGF/c-MET autocrine signaling promoted prostasphere formation [67]. Our study nicely extends these observations to a more relevant experimental setting involving organoid formation by primary cultures of sorted cells enriched in progenitors.

The EGFR/ERBB4, MET and IGF-1R families of growth factor receptor have been implicated in prostate cancer [31,68,69]. Receptors of the EGFR/ERBB family are tightly correlated with poor prognosis, drug resistance, cancer metastasis, and lower survival rate of prostate cancer patients [68,70]. Overexpression of ligands and/or receptors of ERBB and MET pathways has been reported in prostate cancer [69,71,72]. EGFR signaling promotes prostate cancer cell invasiveness and metastasis by inducing epithelial-to-mesenchymal transition (EMT) [73,74], resistance to chemotherapy [75,76] and, ultimately, disease relapse [68,77]. MET is co-expressed with stem-like markers in the invasive cell front of prostate cancer [78], and MET signaling also promotes prostate tumorigenesis [79], invasiveness [80] and migration [81]. Notably, as MET expression is negatively regulated by androgen signaling, androgen deprivation therapy further increases these MET signaling-mediated effects [82,83]. Together, these data demonstrate the pathological relevance of these three receptor pathways identified in silico as top candidate regulators of LSC^med^ cells. Despite this evidence, monotherapies targeting EGFR [22,23], MET [24,25,26,27,28] and IGF-1R [29,30,31] signaling showed limited objective clinical responses in metastatic CRPC patients. Based on the mechanisms of resistance observed in other cancers in which these drugs are used, e.g., non-small cell lung cancer (NSCLC) and head and neck cancer [32,33,34], the therapeutic failure observed in CRPC patients has been generally assumed to be due to the functional redundancy of EGFR/ERBB, MET and IGF-1R, which activate largely overlapping signaling pathways including Ras-Raf-MAPK and PI3K/AKT/mTOR pathways [72,84]. To the best of our knowledge, however, this functional redundancy has not been experimentally assessed in relevant models of CRPC. By showing that LSC^med^ progenitor cells enriched from Pten-null prostates can evade receptor-targeted monotherapies when stimulated by alternative growth factors known to be present in the prostate microenvironment, our preclinical data support such a mechanism in CRPC patients.

In the absence of growth factors in culture medium, virtually no organoids are generated from WT LSC^med^-like cells (our data, not shown, and Ref. [15]). In contrast, in such a medium, Pten-null LSC^med^ cells maintained the ability to generate ~35% of organoid forming capacity compared to EGF-supplemented conditions. This residual property presumably accounts for constitutive PI3K/Akt signaling resulting from *Pten* deletion. Increased cell-autonomous AREG/EGFR signaling in Pten-null LSC^med^ cells may also contribute as the expression of both actors was increased compared to their WT counterparts. Supporting such a mechanism, Erlotinib and Afatinib had a more pronounced inhibitory effect on organoid formation (>90% inhibition) than omission of EGF in culture medium (~65% inhibition). In the latter condition, and even more in the presence of Afatinib, the organoids were of smaller size. This suggests that EGFR signaling not only activates LSC^med^ cell progenitor capacities, but also contributes to their proliferation. In our hands, IGF-1 was as potent as EGFR ligands to stimulate Pten-null LSC^med^ organoid-forming capacity. The observation that IGF-1 was unable to induce organoid formation by WT LSC^med^-like cells [15] suggests the potentiating role of PI3K/Akt signaling in this assay. In situ, IGF-1 is primarily produced by prostatic stromal cells. In human prostate cancer, it was recently suggested that periprostatic adipose tissue promotes resistance to docetaxel by paracrine IGF-1 upregulation, further supporting its role in the tumor microenvironment [85]. Together, our data identify these growth factors as paracrine/autocrine active activators of the progenitor and growth properties of tumoral LSC^med^ cells.

The failure of receptor-targeted monotherapies in metastatic CRPC patients has encouraged the development of combined therapeutic approaches. Combination of EGFR tyrosine kinase inhibitors (gefitinib) and mTOR inhibitor (everolimus) did not result in significant antitumor activity in metastatic CRPC patients [86]. This may be due to relief of the negative feedback of PI3K signaling on AR activity [87], which has supported studies combining PI3K pathway inhibitors and second-generation AR inhibitors in CRPC (https://clinicaltrials.gov/, accessed on 5 July 2022: NCT04737109 and NCT01485861). A recent phase I/II on 44 patients suggested that cabozantinib could be safely added to docetaxel with possible enhanced efficacy [88]. Additionally, ongoing trials are currently recruiting using cabozantinib in combination with abiraterone (CABIOS trial, NCT04477512). Despite these encouraging perspectives, the multiple crosstalk between EGFR, IGF-1R and MET signaling highlighted in preclinical and clinical studies, and herein assessed in castration-tolerant prostate cancer cells, call for concomitant targeting of these three receptors. This is challenging and could lead to additive clinical toxicities for patients. An important outcome of future investigations will be the identification, providing it exists, of a common signaling target downstream of these receptors in order to prevent resistance to receptor-specific monotherapies.

## 5. Conclusions

Based on their intrinsic castration tolerance and stem/progenitor-like properties, prostatic luminal progenitor cells are emerging as important actors of prostate cancer progression. In this study, we report two important findings that improve our understanding of these cells:

First, we demonstrate that LSC^med^ cells isolated from mouse prostate are molecularly equivalent to luminal progenitor and Club/Hillock cell clusters identified by scRNA-seq in mouse and human prostate, respectively. Bridging such in silico transcriptomic information with functional characterization of FACS-enriched luminal progenitor cells knowing they apply to the same cell entity should speed up our understanding of their biology in health and disease. In keeping with this, the common 15-gene signature of LSC^med^-like cells that we provide should help tracing luminal progenitor cells in preclinical models of prostate tumor progression.

Second, we identified EGFR, MET and IGF-1R as regulators of LSC^med^ cells, irrespective of the androgen context. Their functional redundancy in the organoid assay offers several alternatives to circumvent targeted receptor inhibition. These findings (i) may highlight some mechanisms of resistance to strategies targeting these receptors in prostate metastatic CRPC patients, and (ii) call for the identification of common downstream signaling targets to efficiently eradicate these castration-tolerant, cancer-initiating progenitor cells.

## Figures and Tables

**Table 1:** References of scRNA-seq datasets re-analyzed in this study.

Species	Repository	Accession	Data Format	Cell Count (Starting)	Cell Count (Retained)	Ref.
Mouse	GEO	GSE145861 GSE145865	Count matrices in h5 format for each sample	90,345	45,432	[17]
Mouse	GEO	GSE146811	Pooled Count matrices in h5 format	13,688	5158	[15]
Mouse	NODE	OEP000825	Raw fastq files	34,444	19,503	[10]
Mouse	GEO	GSE150692	Raw counts matrices in tsv format for each sample	5288	2362	[16]
Mouse	GEO	GSE151944	MULTI-seq outputs as raw count matrices per sample	4624	1213	[14]
Mouse	GEO	GSE164858	CellRanger output (barcodes, features, matrix files)	6097	2526	[18]
Human	GEO	GSE145843	Pooled Count matrices in h5 format	71,978	28,759	[17]
Human	GEO	GSE150692	Raw counts matrices in tsv format for each sample	6728	3352	[16]
Human	GEO	GSE141445	Single raw counts matrix in txt format for all samples	36,423	24,203	[21]
Human	GEO	GSE176031	Raw counts matrices in txt format for each sample	26,807	14,937	[20]

## Data Availability

The scRNA-seq datasets re-analyzed in the scope of this project were stored as processed R objects in rds format and are available from the corresponding author on reasonable request.

## Supplementary Materials

The following supporting information can be downloaded at: https://www.mdpi.com/article/10.3390/cancers14153775/s1. Figure S1: Gene set enrichment analysis based on functional annotation of genes expressed in luminal progenitor cell clusters identified in scRNA-seq studies, Figure S2: Transcriptomic similarity between FACS-enriched WT mouse LSC^med^ cells and the Lum C1 cell cluster identified by scRNA-seq analysis of prostate tumors of Pten^(i)pe−/−^ mice, Figure S3: Expression of ligand and receptors of the EGFR/ERBB4, MET andIGF-1R families in WT LSC^med^-like cell clusters (scRNA-seq), Figure S4: Expression of the EGFR/ERBB4, MET and IGF-1R ligands and receptors in the various prostate cell populations of WT mouse prostates, Figure S5: Comparative gene expression of the EGFR/ERBB4, MET and IGF-1R ligands in the various prostate cell populations of WT and Pten-null prostates, Figure S6: Impact of various EGFR/ERBB4, MET and IGF-1R ligands on the number and size of organoids and on cell size in organoids generated from PTEN-null LSC^med^ cells in culture medium containing or not DHT, Figure S7: Growth factor stimulation can rescue organoid formation by PTEN-null LSC^med^ cells in the presence of high concentration of Afatinib, Table S1: Primers used for RT-qPCR, Table S2: Media, additives, growth factors and inhibitors used for organoid assays, Table S3: Marker gene identification for the mouse luminal progenitor cluster in scRNA-seq studies (EXCEL file).
