# Supplementary material for: Transcriptomic Signature and Growth Factor Regulation of Castration-Tolerant Prostate Luminal Progenitor Cells
Source: Cancers (Basel). 2022 Aug 3;14(15):3775. doi: 10.3390/cancers14153775 (PMC9367377; doi:10.3390/cancers14153775)
Supplement: Supplementary file 1 [file cancers-14-03775-s001.zip › Supplemental Figures.pdf]

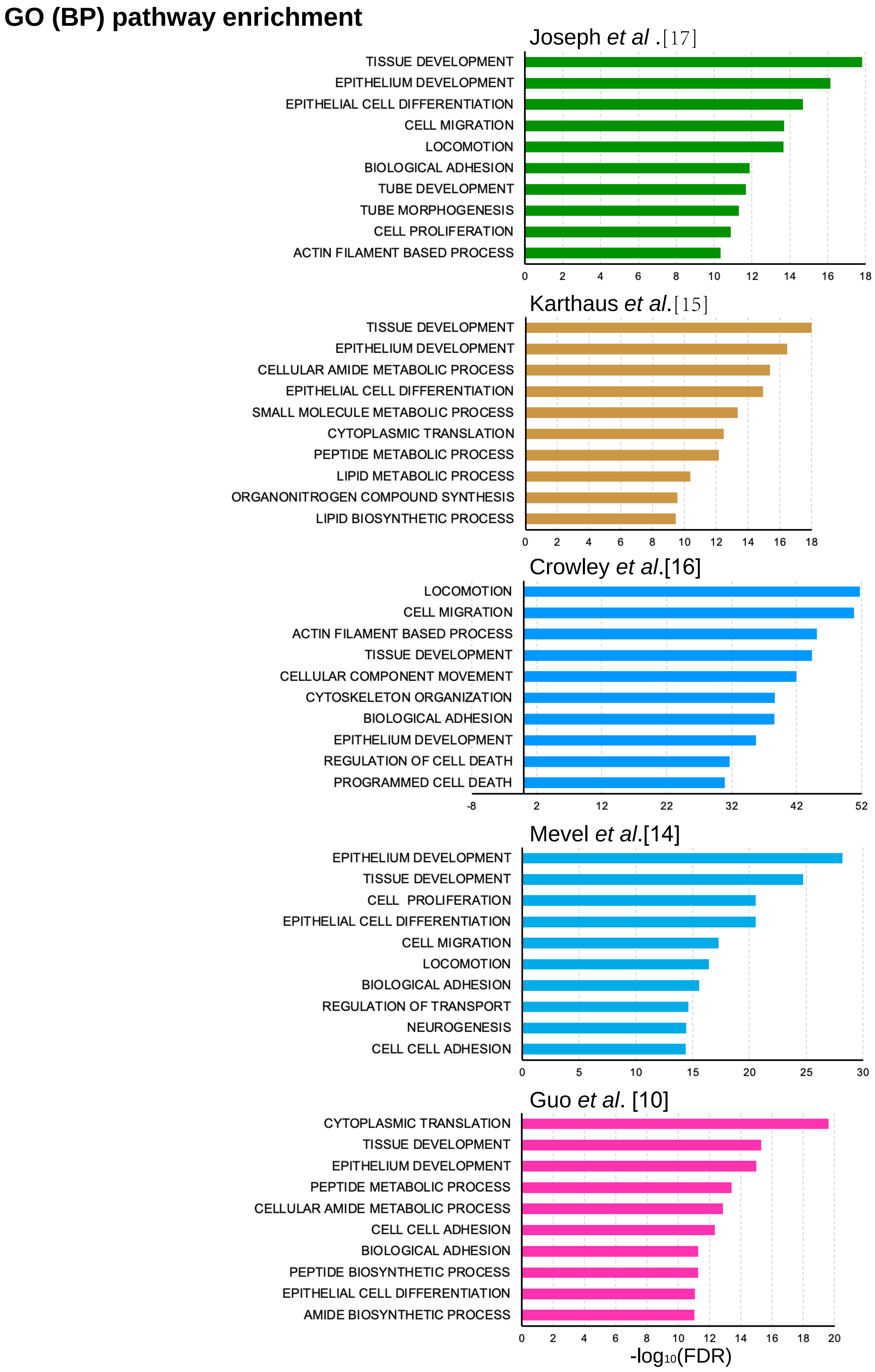

Figure S1. Gene set enrichment analysis based on functional annotation (Gene Ontology—Biological Process database) of genes expressed in luminal progenitor cell clusters identified in scRNA-seq studies. Complementary to Figure 1.

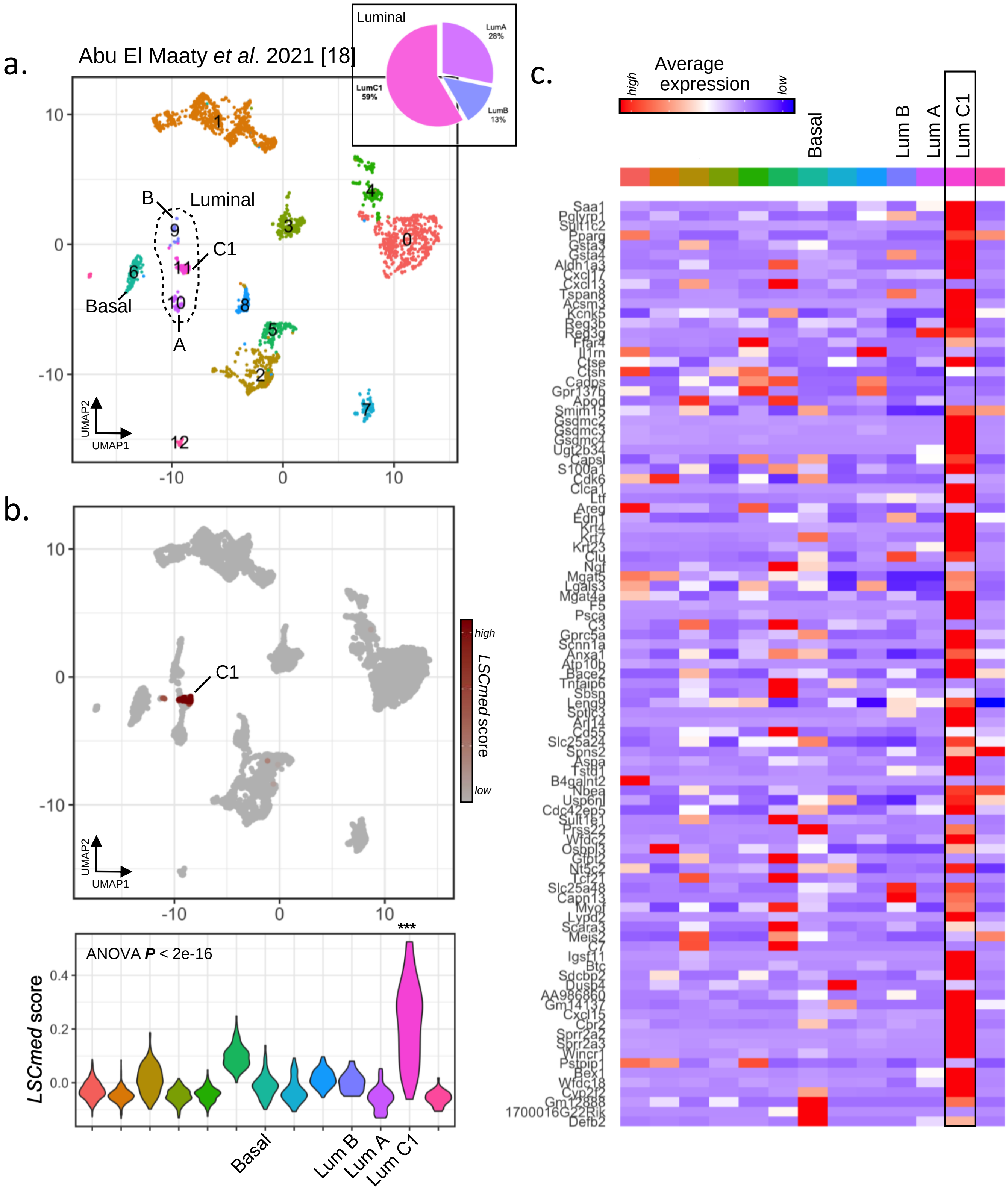

Figure S2. Transcriptomic similarity between FACS-enriched WT mouse LSC^{med} cells and the Lum C1 cell cluster identified by scRNA-seq analysis of prostate tumors of Pten$^{(i)pe-/-}$ mice (vehicle-treated sample in [18]). In this model, ablation of *Pten* is induced at adulthood selectively in luminal prostatic epithelial cells via tamoxifen-dependent CreER^{T2} recombinase under the control of the human PSA promoter. (a) UMAP projection based on linear dimensionality reduction by principal component analysis (PCA) for 2,526 single-cell transcriptomes. The pie chart shows the relative proportions of each Luminal cell sub-population. The prevalence of LumC1 cells is slightly lower than that of LSC^{med} cells in Pten-null mice used in our study. (b) The sole Lum C1 subpopulation matched LSC^{med}-like cells, as shown by high calculated LSC^{med} gene signature scores. The violin plots show the calculated LSC^{med} score per cluster (***, Tukey multiple comparisons of means Padj < 0.001 for all pairwise comparisons performed). (c) Heatmap representation of average expression levels per cluster of LSC^{med} signature genes found in the [18] dataset. Complementary to Figure 1.

a. Receptors

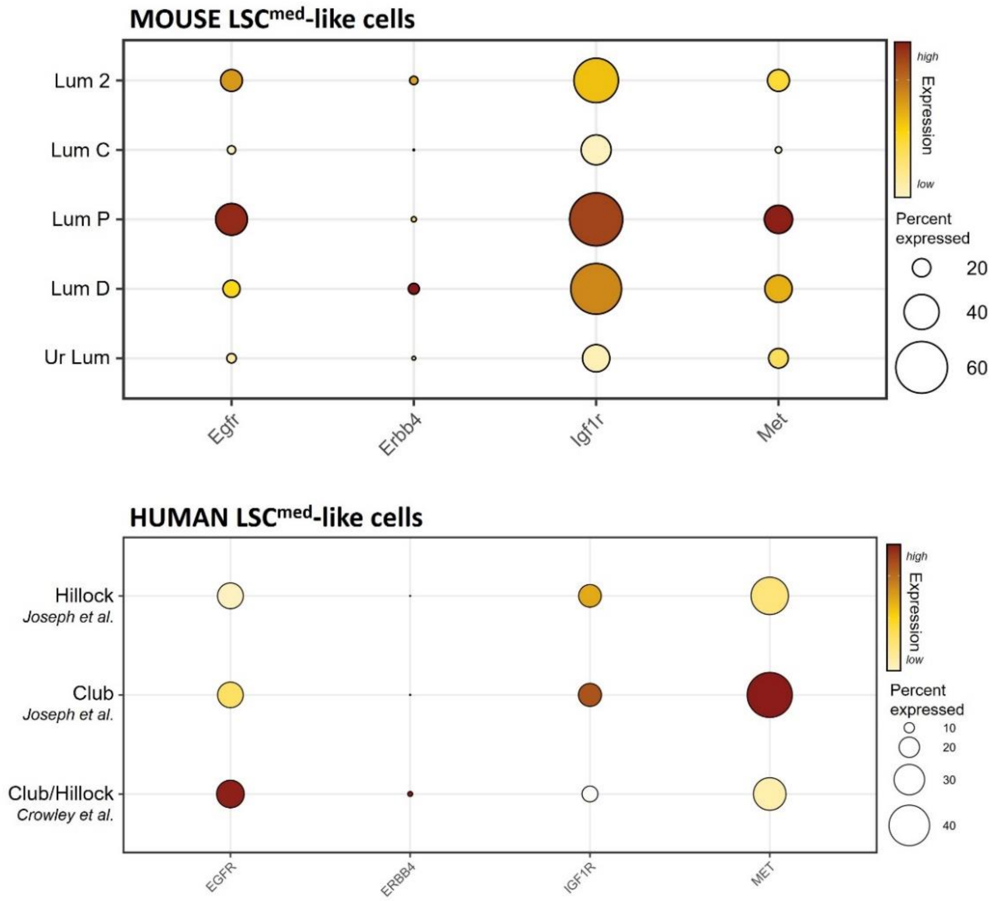

b. Ligands

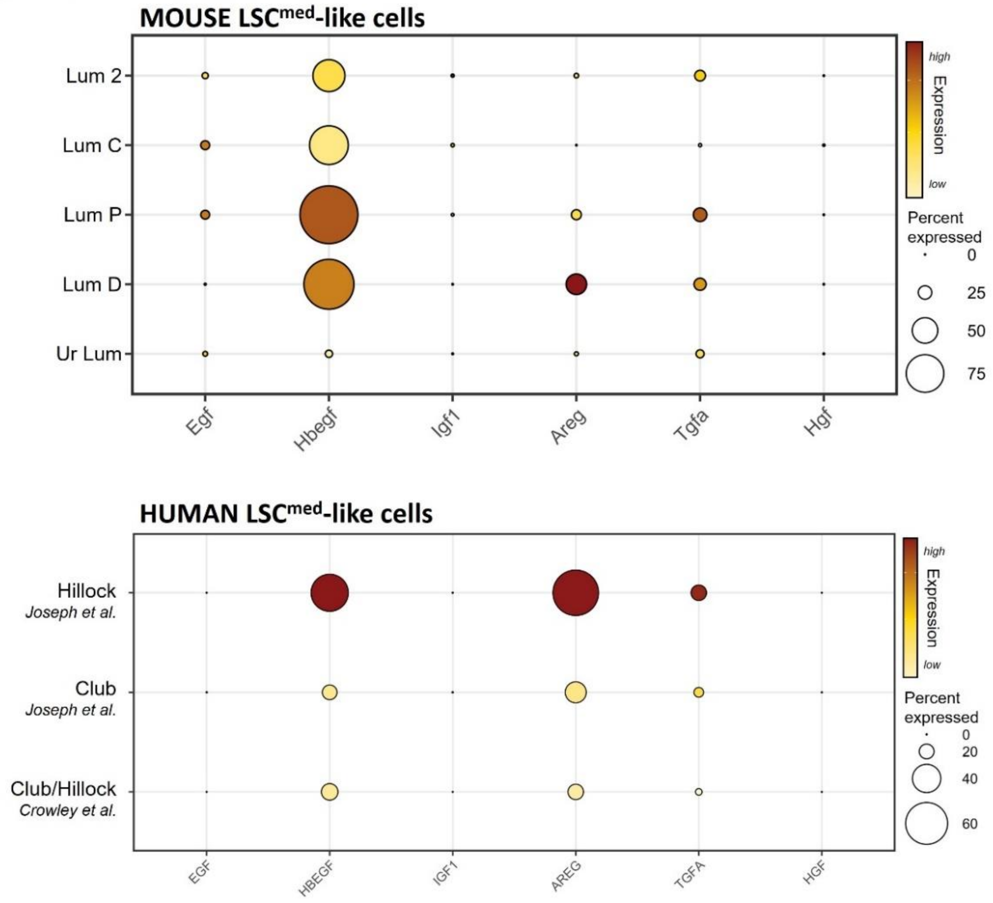

Figure S3. Expression of ligand and receptors of the EGFR/ERBB4, MET and IGF-1R families in WT LSC^{med}-like cell clusters (scRNA-seq). Dot plot representation of the relative expression of genes encoding for (a) receptors or (b) ligands in LSC^{med}-like clusters identified in either mouse or human scRNA-seq assays. Each dot depicts both detection rate (dot diameter) and average gene expression (color scale) in detected cells for a gene in a cluster. Complementary to Figure 4.

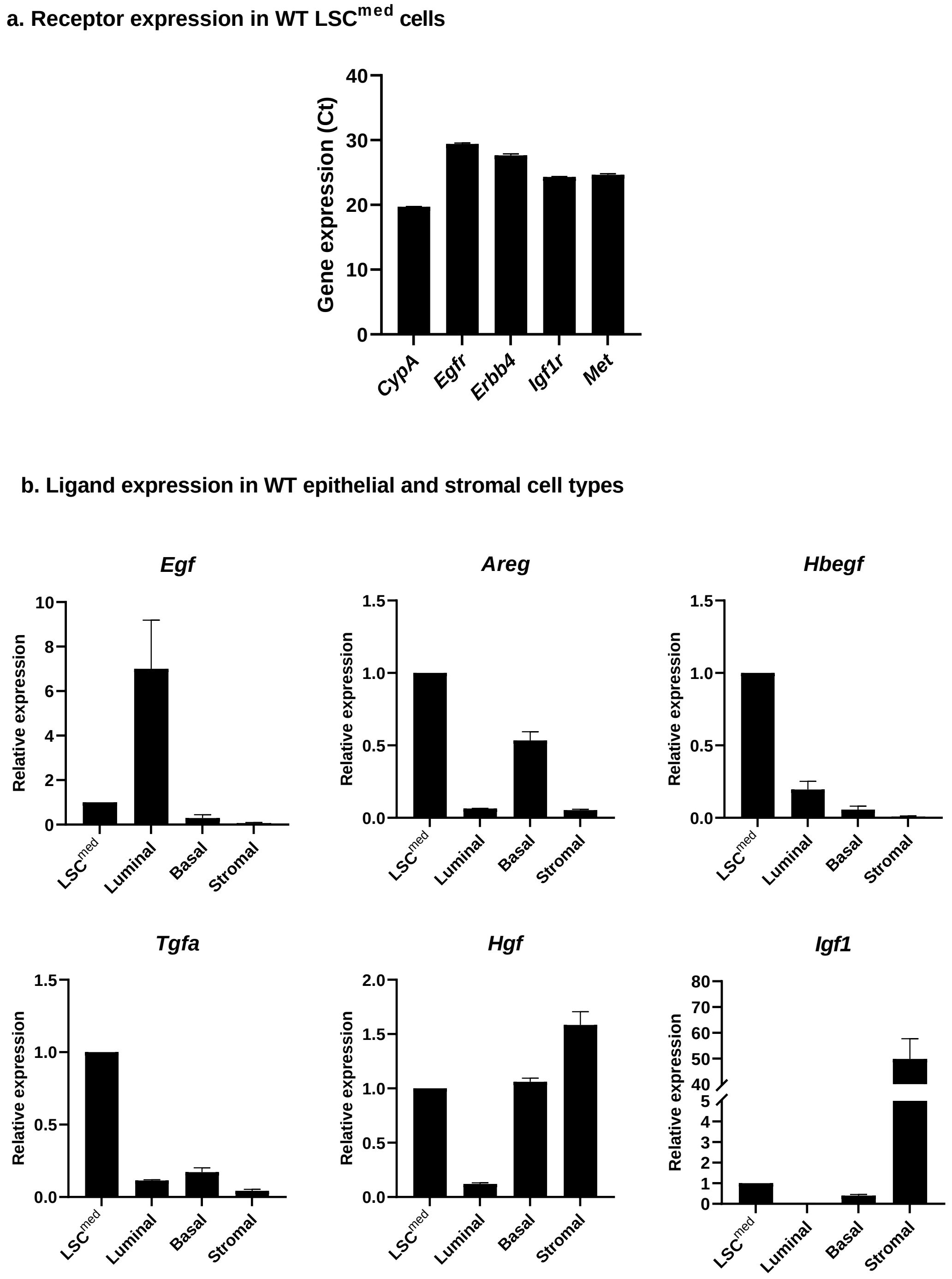

Figure S4. Expression of the EGFR/ERBB4, MET and IGF-1R ligands and receptors in the various prostate cell populations of WT mouse prostates. Expression of *Egfr, Erbb4, Met* and *Igf1r* in LSC^{med} cells (**a**), and of their ligands in the various prostate cell compartments (**b**), was determined by RT-qPCR using mRNA extracted from prostate cell-sorted populations of WT mouse prostates. In panel a, semi-quantitative expression data are represented as Ct values (i.e. the cycle of amplification at which amplified cDNA can be detected) for the four receptors of interest and the house-keeping gene (cyclophilin A). In panel b, relative expression data are represented as $2^{-\Delta\Delta Ct}$ normalized to LSC^{med} cell values. Data were obtained from 3 to 15 animals according to the proportion of the cell type of interest (LSC^{med} cells are less prevalent than other epithelial cell types). Complementary to Figure 5.

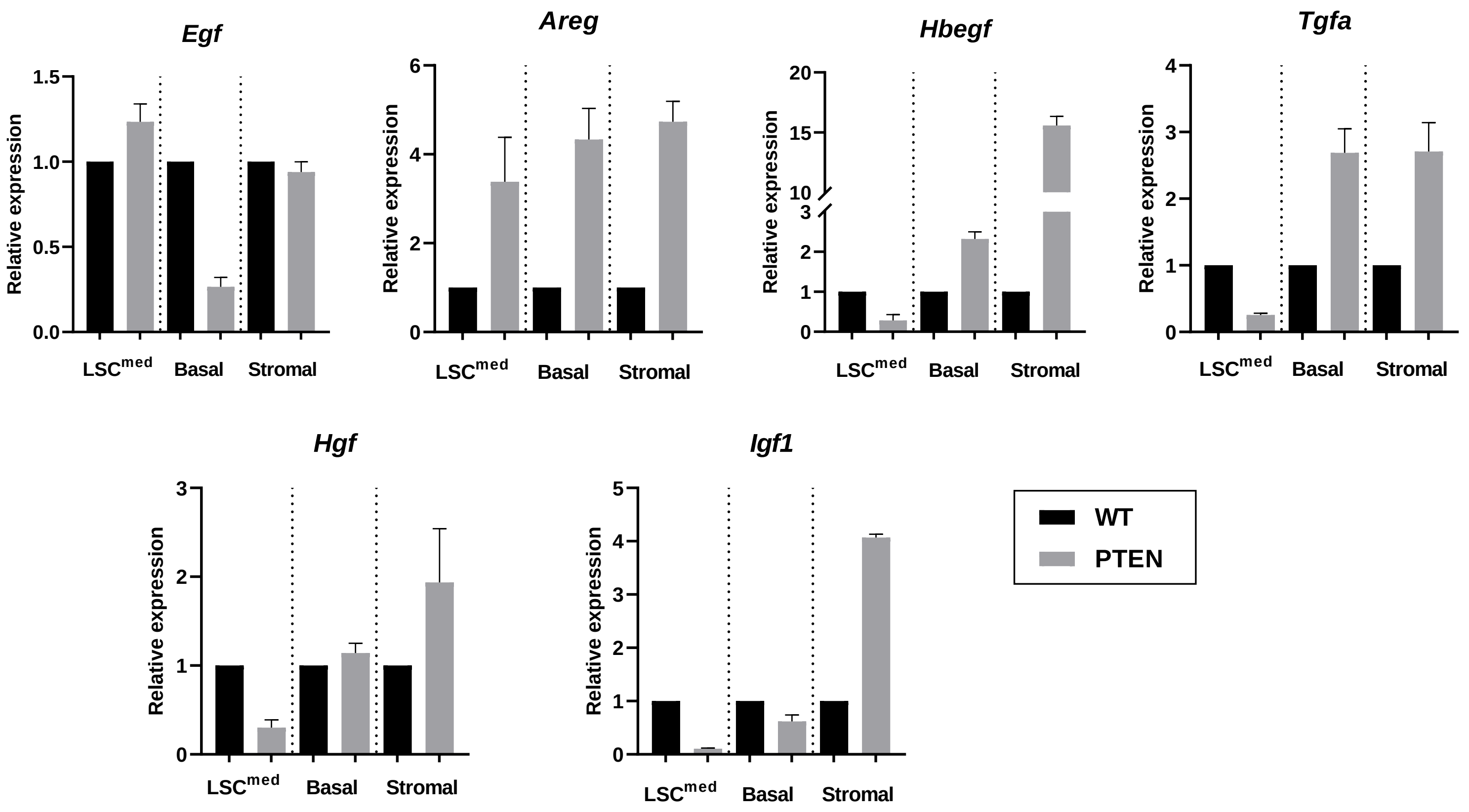

Figure S5. Comparative gene expression of the EGFR/ERBB4, MET and IGF-1R ligands in the various prostate cell populations of WT and Pten-null prostates. Expression was determined by RT-qPCR using mRNA extracted from prostate cell-sorted populations of WT and Pten-null mice, as indicated. Luminal cells could not be included in the analysis due to too low prevalence in Pten-null mouse model. For each cell type, $2^{-\Delta\Delta Ct}$ of Pten-null mice are normalized to WT mouse values. Data were obtained from 3 to 15 animals according to the proportion of the cell type of interest in each genotype. Complementary to Figure 5.

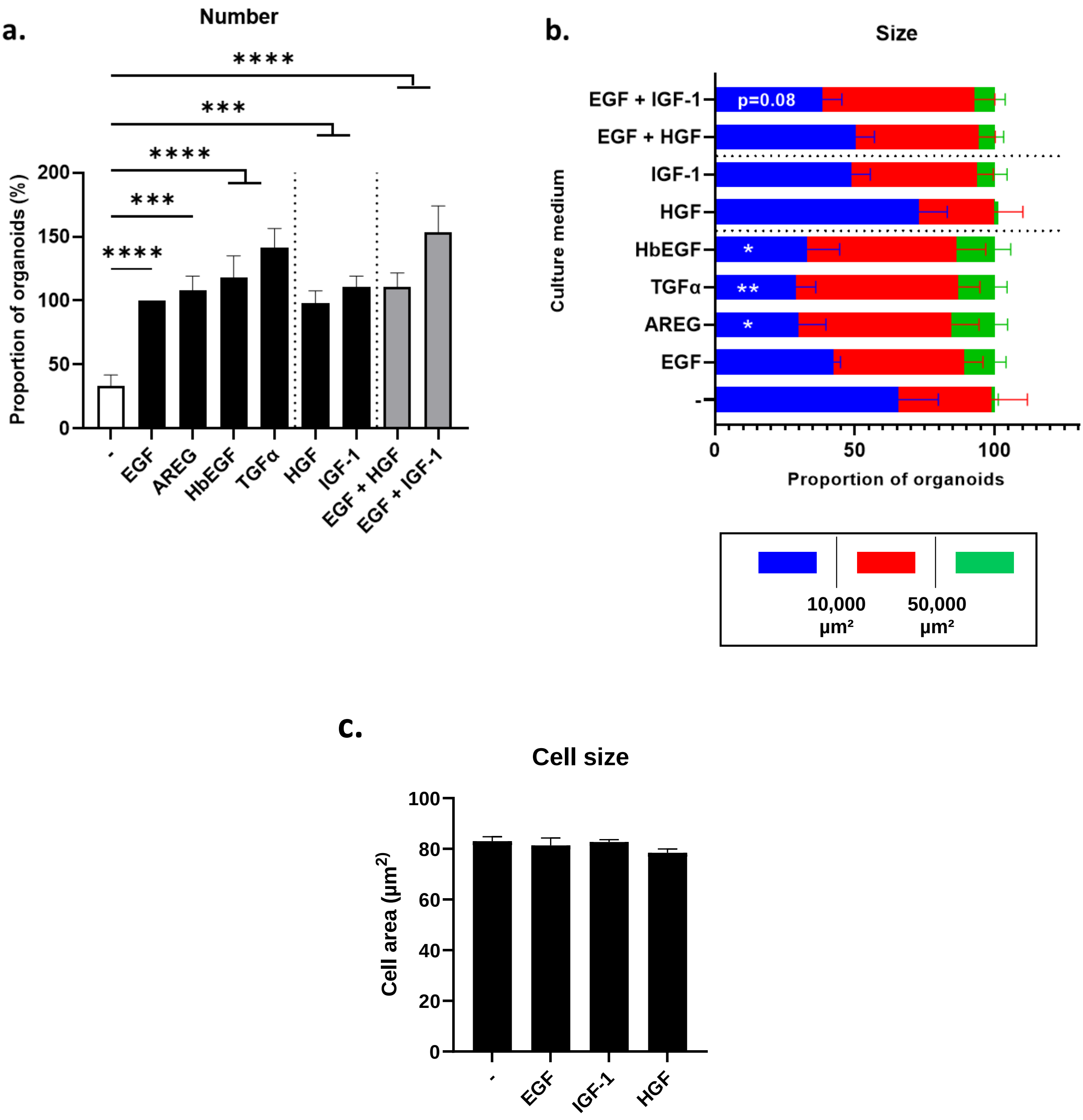

Figure S6 . Impact of various EGFR/ERBB4, MET and IGF-1R ligands on the number (a) and size (b) of organoids and on cell size in organoids (c) generated from PTEN-null LSC^{med} cells in culture medium containing (c) or not (a,b) DHT. See Table S2 for the concentrations of each growth factors added to the culture medium. Data were obtained from 3 independent experiments each involving 1 or 2 animals. Statistical analyses were performed one-way ANOVA followed by Dunnett's post hoc test (a) or two-way ANOVA (b). *p< 0.05; **p <0.01; *** p <0.001 and ****p <0.0001 versus the condition without growth factor (noted as "-"). Complementary to Figure 7.

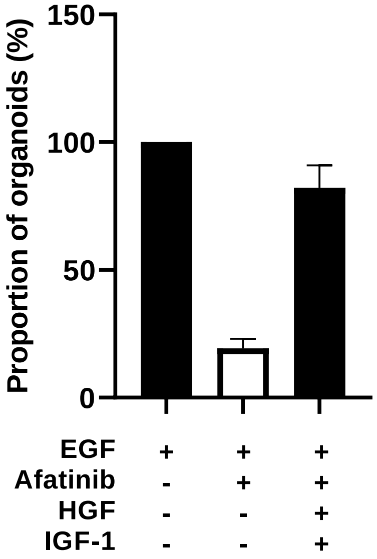

Figure S7 . Growth factor stimulation can rescue organoid formation by PTEN-null LSC^{med} cells in the presence of high concentration of Afatinib (100 nM). This indicates limited drug toxicity. Complementary to Figures 8 and 9.
