# Supplementary material for: Transcriptomic Signature and Growth Factor Regulation of Castration-Tolerant Prostate Luminal Progenitor Cells
Source: Cancers (Basel). 2022 Aug 3;14(15):3775. doi: 10.3390/cancers14153775 (PMC9367377; doi:10.3390/cancers14153775)
Supplement: Supplementary file 1 [file cancers-14-03775-s001.zip › Supplementary Tables.pdf]

Table S1. List of primers used for qPCR.

Target genes	Forward (F) Reverse (R)	Sequence
Cyclophilin A	**F**	CAG-GTC-CTG-GCA-TCT-TGT-CC
	**R**	TTG-CTG-GTC-TTG-CCA-TTC-CT
Egfr	**F**	GCA-TCA-TGG-GAG-AGA-ACA-ACA
	**R**	CTG-CCA-TTG-AAC-GTA-CCC-AGA
Met	**F**	CTG-CAA-ACG-CAG-AAG-TTC-ACC
	**R**	TGT-TCA-CGT-CGG-GAT-AAG-GAG
Igf1r	**F**	TTC-AGT-TCG-TGT-GTG-GAC-CGA-G
	**R**	TCC-ACA-ATG-CCT-GTC-TGA-GGT-G
Egf	**F**	GGC-TCT-TCT-GGG-TTC-AGG-AC
	**R**	CAA-ACT-GTG-CCG-TGC-TTG-AT
Areg	**F**	GGT-CTT-AGG-CTC-AGG-CCA-TTA
	**R**	CGC-TTA-TGG-TGG-AAA-CCT-CTC
Hbegf	**F**	CGG-GGA-GTG-CAG-ATA-CCT-G
	**R**	TTC-TCC-ACT-GGT-AGA-GTC-AGC
Tgfa	**F**	CAC-TCT-GGG-TAC-GTG-GGT-G
	**R**	CAC-AGG-TGA-TAA-TGA-GGA-CAG-C
Hgf	**F**	GAG-TTA-TGT-GCT-GGG-GCT-GAA
	**R**	CGA-CCA-GGA-ACA-ATG-ACA-CCA
Igf1	**F**	TTC-AGT-TCG-TGT-GTG-GAC-CGA-G
	**R**	TCC-ACA-ATG-CCT-GTC-TGA-GGT-G

Table S2. Media, additives, growth factors and inhibitors used for organoid assays.

	Component	Company	Concentration
**Media and additives**	Advanced DMEM/F12 medium	Gibco	
	Pen/Strep Antibiotics	Gibco	1%
	Glutamax	Gibco	1%
	Hepes	Gibco	1%
	B27 (minus Vitamin A)	Gibco-Invitrogen	2%
	NAC	Sigma-Alrich	1.25 nM
	Y-27632	Sigma-Alrich	10 nM
	Noggin	Peprotech	100 ng/mL
	R-Spondin	Peprotech	500 ng/mL
	A83.01	Sigma-Alrich	200 nM
	5-Dihydrotestosterone (5-DHT)	Sigma-Alrich	1 nM
**Growth Factors**	EGF	Peprotech	50 ng/mL
	AREG	Peprotech	200 ng/mL
	HB-EGF	Peprotech	50 ng/mL
	TGFα	Peprotech	50 ng/mL
	HGF	Peprotech	50 ng/mL
	IGF-1	Peprotech	200 ng/mL
**Drugs**	Afatinib	MedChemExpress	0.1-100 nM
	Erlotinib	MedChemExpress	0.1-100 nM
	Cabozantinib	MedChemExpress	0.1-100 nM
